# Combining exploration and imitation in contact-rich task learning on an articulated soft robot arm

**DOI:** 10.3389/frobt.2026.1885625

**Published:** 2026-08-05

**Authors:** Laurenz Elstner, Erik Kyrkjebø, Martin F. Stoelen

**Affiliations:** 1 Department of Computer Science, Electrical Engineering and Mathematical Sciences, Western Norway University of Applied Sciences, Førde, Norway; 2 Fieldwork Robotics Ltd., Cambridge, United Kingdom

**Keywords:** contact-rich task learning, human-inspired robotics, imitation learning, learning from demonstration, reinforcement learning, soft robotics, variable-stiffness actuated system

## Abstract

Learning from demonstration (LfD) has become a popular approach with the emergence of modern transformer-based algorithms. However, the performance of these policies is limited by the quality of the demonstrations. Combining imitation and exploration promises to train policies that perform better and are more reliable. However, this requires a robotic system that can explore safely without damaging itself or the environment, especially in contact-rich tasks during which the robot must exert force on its environment to solve the task. In this study, we investigate the combination of a state-of-the-art reinforcement learning (RL) algorithm with human demonstrations to learn how to open a door with minimal task-specific engineering on an articulated soft robot arm. We found that learning from both exploration and demonstration data stored in separate buffers makes the algorithm not only more sample-efficient and robust but also allows the policy to reach a higher performance level than the provided expert demonstrations. We also show that using an articulated soft robotic arm allows us to perform RL on a real robotic system without any pretraining and with a simple safety system that does not require any additional sensors, such as force–torque sensors. Additionally, we can implicitly learn the nonlinearities stemming from the soft materials in the actuator. Our findings show that combining LfD with RL results in both better performance and more robust behaviors and indicate that articulated soft robots allow for learning contact-rich tasks safely on a real system.

## 1 Introduction

Humans learn to solve a wide variety of tasks using a combination of imitation and exploration (Grimes and Rao, 2009). Imitation is the process of learning by observing an expert solve a task and attempting to copy that movement. However, because each person’s morphology is different, exploration is also required to optimize movement. Exploration means trying something new without knowing the results. To explore safely, a robot requires some form of softness. Collaborative robots (cobots) achieve this softness by using force-torque sensors in a control loop to simulate spring-like behavior. Because the softness is simulated, the spring stiffness properties can be adjusted during runtime. The problem is that if the impact is too fast for the controller to react in time, it will be as hard as if there were no softness. This is one of the reasons why cobots have a lower maximum velocity than industrial robots. Another approach to achieve compliant behavior is to use soft materials. Robots with continuously deformable structures are considered continuum soft robots; robots with rigid kinematic structures but soft materials in the actuation of the joint are categorized as articulated soft robots (Angelini et al., 2018). Articulated soft robots can often vary the stiffness of their joints using variable-stiffness actuators (VSA), which typically require two motors per joint.

**FIGURE 1:**
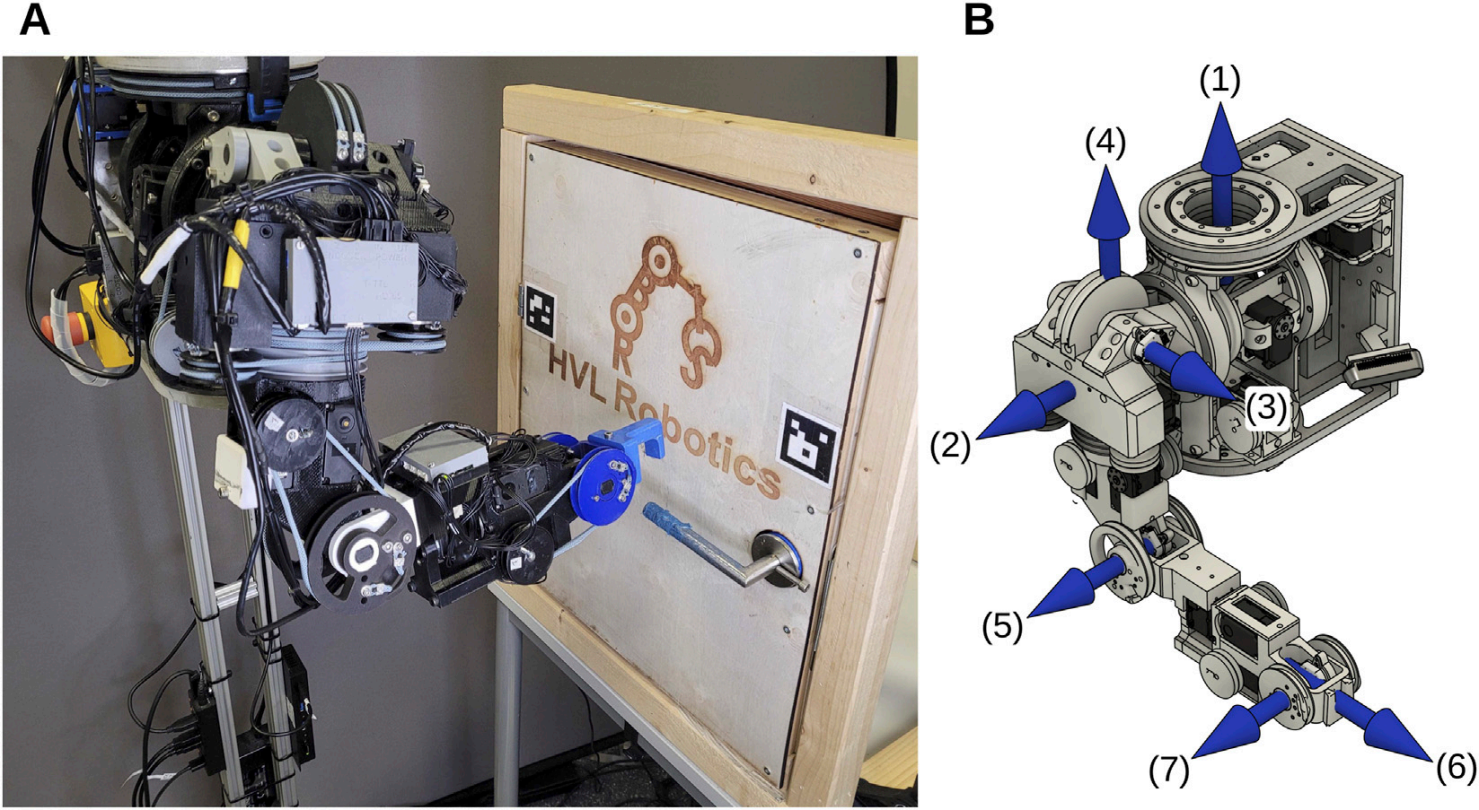
**(A)** GH360 in the door environment. **(B)** Joint structure: (1) shoulder yaw, (2) shoulder roll, (3) shoulder pitch, (4) upper-arm roll, (5) elbow, (6) forearm roll, and (7) wrist pitch. Six of seven joints are variable-stiffness actuators (VSAs); the forearm roll is directly driven by one motor.

In the simplest setup, one motor controls the joint position, and another motor changes the joint’s stiffness by tensioning a nonlinear spring. Here, the maximum joint torque is limited by the joint position motor. However, the second motor can be comparatively small because it only needs to tension the spring. The MACCEPA 2.0, used for legged locomotion, uses this mechanism (Vanderborght et al., 2009). Zhu et al. (2022) introduced extra pulleys to increase the maximum torque and resolution of the stiffness variation. Another common type of VSA uses two motors in an agonistic/antagonistic setup. Here, the motors move together to change the joint position and in opposite directions to vary the stiffness. Because both motors are used for stiffness and position changes, they must be equal in strength. If each motor is only connected to one side of the joint, the maximum joint torque is constrained by the torque of a single motor. In a bidirectional setup, however, the maximum torque is limited by the combined strength of both motors. Examples of systems that have implemented this type of actuator include the DLR hand arm system (Grebenstein et al., 2011), Vsa-CubeBot (Catalano et al., 2011), qbmove Advanced II (Mengacci et al., 2021), and the GH360 robot used in this study, shown in Figure 1.

Although the soft material allows the robot to absorb unexpected and highly dynamic events, it also increases control complexity because of its nonlinear behavior and hysteresis effect. Because articulated soft robots have a rigid kinematic structure, many model-based control strategies from classical robotic control can still be applied. However, Santina et al. (2017) argued that classic high-gain feedback control strategies fight against the compliance introduced into soft robots to achieve good trajectory tracking by effectively stiffening them. This has resulted in the emergence of learning-based controllers. Angelini et al. (2018) use a combination of low-gain feedback and iterative learning control (ILC) in the feedforward component to cope with the complex dynamics of the soft material. Hofer et al. (2019) use a norm-optimal ILC (NOILC) approach in parallel with a PID feedback controller to control a joint that uses two soft bellow actuators in an antagonistic setup. Pierallini et al. (2023) use a purely feedforward ILC algorithm to achieve good trajectory tracking on a soft continuum robot.

Feedback controllers can only react to inputs and disturbances and therefore have a lag. Feedforward control does not have this lag but requires an accurate model of the system and is not robust against disturbances. The basic idea behind ILC is that it learns to anticipate and compensate for repeating disturbances, and, in the case of soft robotics, model errors due to the nonlinearities and hysteresis effects inherent in soft materials. However, it is designed to be used for repeating the same trajectory and will need to relearn whenever a new trajectory is executed. Veronese et al. (2024) learn a data-driven feedforward model and use a feedback controller to both correct the output of the feedforward controller and use it as a learning signal to train/adapt the feedforward model. The feedback controller is a high-gain PID, which, as described previously, contradicts the use of a soft robot. Büchler et al. (2018) learn a probabilistic dynamics forward model of an arm with pneumatic artificial muscles (PAMs). During operation, the Gaussian process dynamics model is used in feedforward control.

A higher-level approach to using machine learning is to learn a task through exploration, that is, learning from trial and error. This concept is referred to as reinforcement learning (RL) and has proven to be powerful in solving complex tasks. In RL, a policy is trained to take actions given observations that partially represent the state of the robot and its environment. The policy starts in an untrained state, meaning that it will start by sending random commands to the robot controller. A reward system provides more or fewer rewards depending on whether the action was good or bad in terms of solving the task. Rewards are used as reinforcement signals to train the policy to select actions that will result in the maximum amount of long-term rewards.

Modern algorithms often use an actor-critic structure, in which the critic is trained to predict the potential long-term rewards of an action in a given state. This value estimate is used to train the actor, which chooses actions given the current state. Therefore, the reward function determines the robot’s behavior. A reward function can either be sparse, meaning that the only reward given is once the task has been successful, or dense, in which intermittent rewards are given to guide the policy to a successful state. For example, when learning to open a door, a sparse reward function would provide full rewards when the door is open and no rewards otherwise. However, a dense reward function might provide increasing rewards when the end-effector (EEF) gets close to the door handle, when the door handle is pressed down, and when the door is being opened. Sparse rewards are easier to define but are much more difficult for the RL policy to learn because they require random movements to successfully execute the task to receive a learning signal. Dense reward functions must be carefully engineered because they can reinforce unwanted behaviors.

In deep RL, the policy is represented by a neural network, which in most cases is a simple feedforward neural network, also known as a multilayer perceptron (MLP). The network parameters, such as the weights of the connections between neurons, are adjusted using the training data. Neural networks and RL algorithms also have parameters that are set manually, for example, the learning rate or how many neurons the network contains. These are called hyperparameters. Satheeshbabu et al. (2019) learn a policy that can do accurate trajectory tracking using an open-loop controller on a pneumatically actuated soft continuum arm. Or et al. (2023) train a tendon-driven, high-degrees of freedom (DoF) manipulator inspired by the ostrich to do free-space reaching tasks even under the disturbance of a moving mobile base using a curriculum RL approach. However, this approach was only evaluated in simulations. Büchler et al. (2022) learn to play table tennis with a robot arm driven by PAMs. To reduce the amount of training required, the authors combined learning on the real robot (more accurate data) with learning in simulation (faster learning and easier to automate).

An RL algorithm requires large amounts of data to be successful. That amount of data is expensive to gather on a real robot system, resulting in long learning times. An approach to reduce learning time is to demonstrate the task to the system, thereby guiding the learning towards a solution. LfD, also known as imitation learning, by itself changes the learning goal from RL, that is, trial-and-error learning, to a supervised learning problem, as the goal is to train a policy to produce the same control action in a given state as the demonstrator would do. LfD promises to teach a robot a behavior with minimal overhead, as the idea is that the user simply demonstrates the task to the robot, which in turn learns to imitate the same behavior. This allows a task expert who does not have technical/programming knowledge to teach a robot.

The simplest approach is to represent the policy as an MLP and train it in a supervised manner. This approach is often referred to as behavioral cloning (BC). Mandlekar et al. (2022) show that when learning from human demonstration, history-dependent models perform much better because, when the human gives the demonstration, the decision about what action to do next depends both on the current state of the environment and on the previous states. Therefore, using a recurrent neural network (RNN) and training it in a supervised manner, known as BC-RNN, tends to perform much better, especially as the task becomes more complex.

Newer imitation learning methodologies have evolved from the emergence of LLMs and image generators. Zhao et al. (2023) used a transformer-based architecture in their algorithm called Action Chunking with Transformers (ACT). They train a policy to predict the next sequence of actions, similar to how LLMs predict the next words in a text. It allows them to learn bimanual tasks with a success rate of 80%–90% with only 10 min of demonstrations. Chi et al. (2025) introduce a new policy type that uses the diffusion process found in generating images from noise to instead generate an action sequence in the robot action space. Diffusion policies are robust to multi-modality. For example, they can effectively handle tasks with multiple solution paths or orders in which to perform sub-goals, such as in what order to take out the objects when emptying the dishwasher. This comes with a higher computational cost and inference latency than simpler policies, which is why they predict a sequence of actions but can still be too slow for high-rate control tasks. Zhao et al. (2025) combine the two approaches to train a transformer-based policy with diffusion loss, which they use to learn highly dexterous bimanual manipulation tasks such as tying shoelaces or hanging a shirt on a cloth hanger. However, they require a large amount of data and significant computational hardware. Specifically, they gathered more than 5,000 demonstrations per task and used 64 TPUv5e chips for training, which took approximately 265 h. Even at inference time, the denoising process takes 0.043 s on an RTX 4090 GPU.

In general, when learning only from imitation, the performance of the policy is limited by the quality of the demonstrations. Learning only from exploration, on the other hand, requires much data, especially in a sparse reward environment and when the task horizon and/or action dimensionality increases. In theory, by combining the two approaches, imitation allows the policy to discover high-reward regions faster even when rewards are sparse, and exploration allows the policy to improve further than the performance level given by the expert demonstrations. Gupta et al. (2016) use human demonstrations of dexterous manipulation such as operating an abacus to teach a pneumatically actuated soft robotic hand to do the same. While both RL and LfD are used, the authors use RL to find a control policy that matches the human demonstrations; therefore, at (the very) best, matching the performance of the human demonstrations.

Instead of using RL to imitate demonstrations, Hester et al. (2018) used demonstrations to make a deep RL algorithm more sample-efficient for learning to play different computer games. They achieved this by pretraining the policy of a deep Q-learning algorithm and, after pretraining, learning on a combination of exploration and demonstration data. The ratio between the demonstration and self-generated data was controlled using a prioritized replay mechanism proposed in Schaul et al. (2016). Nair et al. (2018) combine the Deep Deterministic Policy Gradient (DDPG) algorithm from Lillicrap et al. (2016) with 100 human demonstrations in a demonstration buffer to learn a robot arm to stack blocks in simulation. The demonstrations are stored in a separate buffer, with a fixed number of samples added to each mini-batch for training. Additionally, the authors used a BC loss in combination with a Q-filter. That is, if the critic determines that the action of the demonstration is better than the action chosen by the actor, an additional BC loss is added to the actor update.

Another way to use demonstrations to guide exploration is to learn the reward function from them. This is also known as inverse reinforcement learning (IRL). In Ibarz et al. (2018), a combination of demonstrations available before learning and human preferences gathered during training was used to infer a reward function to learn to play Atari games with a deep-Q algorithm. The reward function is represented by a convolutional neural network (CNN), which takes an observation as input and outputs a reward. The reward model is trained by providing the human expert with two video clips showing segments of trajectory rollouts, and the human selects the one that is closer to the desired behavior. This was originally introduced by Christiano et al. (2017).

Ibarz et al. (2018) used demonstrations to auto-label pairs at the beginning of learning by using demonstration trajectories as one of the two clips and automatically labeling them as the preferred behavior. Learning a reward function from demonstrations is highly sensitive to the quality of the demonstrations. Preference-based learning methods can be inefficient because binary feedback is used to learn a continuous reward function. Palan et al. (2019) argue that preference queries are more accurate but less informative than demonstrations. Therefore, they use demonstrations to learn a prior over reward functions, reducing the size of the search space and then using a preference-based system to learn the true reward function within that space. In general, however, these methodologies for learning a reward function rely on both high-quality demonstrations and the user being in the loop during the entire learning process to be effective.

In RL, the robot must execute random actions or modified actions different from those that the policy considers best to explore to possibly find a better behavior. Traditional robots require a sophisticated safety system that can protect both the robot and its environment from collisions or other dangerous actions. Having inherent compliance built into the robot through soft materials can reduce the number of sensors required and the complexity of the safety system.

Simon et al. (2024) show how a robot arm driven by PAMs can have approximately three times as high a contact velocity to produce a similar amount of impact force as a Franka Emika Panda or a Universal Robot UR5e. This is even more important in contact-rich tasks, during which collisions cannot be simply avoided because they are required to solve the task. Contact-rich tasks can be broadly divided into two categories: continuous contact and kinematic constraints. Continuous contact requires the robot to apply a certain amount of force in one direction while generating motion in the other direction, for example, wiping a surface. In a kinematic constraint task, the robot movement is constrained by the degrees of freedom (DoF) of the object it is manipulating, such as opening a door. Chitta et al. (2010) show that kinematic constrained tasks, such as opening a door, are not trivial problems for a rigid robot. Hosoda et al. (2012) use a pneumatically actuated anthropomorphic muscular-skeletal robotic arm to show that physical compliance can reduce the complexity of opening a door, especially once the arm has grabbed the handle and is constrained by the door’s DoF.

Stuede et al. (2019) use a collaborative robot arm with an impedance controller connected to an omnidirectional moving platform to open and move through doors. They used deep learning to detect the door handle, a predefined trajectory to grasp the handle, and hard-coded force values to push the handle down. The robot opens and moves through the door with a combination of three separate controllers. The three controllers for opening the door are as follows: a feedfoward task controller in charge of opening the door with constant velocity, a grasp controller that uses a hybrid structure with a rotational controller and a force controller to ensure that the robot stays in contact with the handle, and a base controller that controls ensures the mobile base of the robot moves continuously through the doorway during the opening process.

Wang et al. (2019) show that physical passive compliance not only reduces the complexity of solving contact-rich tasks but can also eliminate the need for force/torque sensing, as in an example of a peg-in-hole assembly task. Martin-Martin et al. (2019) show that the reduction of complexity through softness also extends to faster and more stable learning of contact-rich tasks. Gu et al. (2017) learn to open a door without demonstrations by introducing an asynchronous version of the NAF algorithm (Gu et al., 2016), which extends deep Q-learning to continuous action spaces. They reduced the learning time by exploring with two robots simultaneously. The robot manipulator is not described or specified but is only referenced as a “7-DoF arm.” A joint velocity controller was used as the action space. In all their experiments, they used joint position and velocity as well as end-effector position constraints. For tasks that require contact, such as opening a door, additional heuristics are required to prevent unsafe exploration, but they do not specify what those heuristics are.

Lin et al. (2021) decompose a task into a sequence of action primitives. For door opening, the primitives are APPROACH DOOR, GRASP HANDLE, and DOOR OPEN. Each primitive was pretrained using offline expert demonstrations and fine-tuned in a simulated environment. Additionally, a label predictor that can identify action primitives inside a trajectory is trained. For each expert demonstration, they predicted the primitives contained in the trajectory to obtain sub-trajectories and executed the predicted action primitives in sequence. The execution of primitives is additionally monitored for safety, meaning that if the current state deviates too much from the expected state or if the allowed time for a primitive is out, the task is stopped, and the primitive is retrained. Otherwise, the authors rely on the built-in protective stop from the UR5e if the motion or contact forces are outside the allowed boundary values.

Zhang et al. (2024) learn a legged robot with a manipulator on top to open a door and walk through it. The authors trained a single policy that could handle both pushing and pulling to open left- and right-sided doors without prior knowledge. The mobile base is controlled by its own policy, taking as input the target linear velocities in x and y and the angular velocity around z, as well as the applied wrench on the robot base from the arm motions. The arm is controlled through joint-level PD controllers, but the computed torque is clipped for safety to not exceed a defined value. Learning is performed using a teacher-student approach. The teacher is trained in simulation using an actor-critic structure, namely, the Proximal Policy Optimization (PPO) (Schulman et al., 2017) algorithm, and has access to additional privileged observations only available in simulation, such as door joint (hinge and handle) positions and velocities, mass of the door panel, door type, torques applied to door joints, and current stage of the task (opening or passing through the door). The student policy is based on a recurrent neural network (RNN) architecture and is trained to imitate the teacher policy given only proprioceptive observations from the robot (base orientation, base linear and angular velocities, arm joint positions and velocities, and previous step actions) and exteroceptive door observations (location of the doorway and handle relative to the robot). After training in the simulation, the student policy was deployed on the real robot. In the reward function, an additional safety term is added that incentivizes a policy that respects the hardware limitations and is safe to deploy on the real robot, such as avoiding collision, penalizing out-of-limit commands, or minimizing arm motions.

In summary, using soft materials provides the robot with passive compliance and allows for the absorption of rapid impacts. However, the nonlinearity and hysteresis effect of this type of material make accurate control much more complex. In a kinematically constrained task, such as opening a door, on the other hand, passive compliance can make it easier to complete the task. Although there is much research on using learning-based controllers for soft robots, full task learning appears to be unexplored. Learning from exploration can be used to learn complex tasks but requires a large amount of data. Imitation learning is much more data-efficient and faster but is highly reliant on the quality of the demonstration data in terms of performance and robustness. Combining the two approaches promises to increase the learning speed while also allowing the robot to learn to perform better than the demonstrator.

In this study, we explore the combination of LfD with RL to learn a contact-rich task on an articulated soft robot arm with minimal task-specific engineering. The manipulator used in this project is the GH360, a human-inspired robot arm with seven DoF, six of which are used in a bidirectional agonistic/antagonistic VSA setup. Learning is performed using a state-of-the-art DRL algorithm, namely, TD7 (Fujimoto et al., 2023), which we modified to allow for hybrid learning from both exploration and imitation. The actions of the policy were sent to an open-loop EEF velocity controller.

We chose to use an operational space controller to reduce the learning complexity. We chose an open-loop controller to avoid working against the inherent compliance of the soft tendons and to allow the policy to learn the model implicitly. We evaluated our proposed algorithm on the door-opening task, which requires a combination of accurate trajectory tracking (moving the EEF into the correct position on the door handle) and kinematically constrained movements while applying force to an object (pushing down the door handle). To the best of our knowledge, no study has been conducted in which full contact-rich task learning was performed on a soft robotic manipulator. We first tested this approach in simulation with an ablation study on the newly added hyperparameters. Subsequently, we tested our proposed algorithm on the real robot.

The main contribution of this work is efficiently learning contact-rich tasks end-to-end on an articulated soft robot arm by combining imitation with exploration and minimal task-specific human engineering/programming. In addition, we extended the TD7 implementation to learn from both exploration and expert demonstration data, as well as adding the possibility of stopping and continuing learning, which is an important feature when performing RL on a real robot. All code and supplementary videos can be found here: https://lauels.github.io/gh360-rl-with-lfd/.

## 2 Materials and methods

### 2.1 Learning from exploration

The simplest implementation of an RL algorithm is called Q-learning. It creates a table of cells denoting every possible state–action pair. The value of each cell, also known as the Q value, represents the expected future rewards and is calculated using the Bellman equation. Although this is effective when the number of states and actions is relatively small, it quickly becomes inefficient or infeasible if the number increases. In robotics, both states and actions are generally continuous, which led to the emergence of neural networks as function approximators for the Bellman equation. This was first introduced by Mnih et al. (2013) with the Deep Q-network algorithm, in which the neural network provides an approximation of the Q value for each possible action given the current observation. Therefore, the network requires one input neuron per state variable. For example, the joint angles of a 7-DoF arm require seven neurons and one output neuron for each possible action. This means that the algorithm can handle continuous observations well but is not suitable for large action spaces, as this would require too many output neurons. Actor-critic methods use two neural networks to solve this. The actor, also known as the policy, maps observations into actions and therefore has one input neuron per observation variable and one output neuron per action variable. The critic produces an estimated reward value given the observation and chosen action. The critic is trained using the reward signal from the environment, and the actor is trained using the estimated reward from the critic.

The DDPG algorithm is an implementation of the actor-critic architecture introduced by Lillicrap et al. (2016). Although DDPG is designed to learn in continuous action spaces, it is known to be brittle in terms of tuning hyperparameters. One common failure point is that the Q-function approximation (critic) overestimates the Q-values of some areas of the action space, which leads the policy to learn to try and exploit those erroneous values. Fujimoto et al. (2018) address this issue in their Twin Delayed DDPG (TD3) by using two critic networks and, at each training step, selecting the minimum value estimate. To improve sample efficiency, Fujimoto et al. (2023) introduced an encoder network that learns the underlying structure and patterns in the state–action data. This technique, also known as feature learning, is commonly used in vision-based environments, where high-dimensional observation spaces, such as images, are compressed into a smaller, more manageable latent vector. The output of the encoder network is fed as an input into both the actor and critic networks.

One way of categorizing RL algorithms is into on-policy and off-policy algorithms. On-policy algorithms use data collected during exploration with the current iteration of the policy for learning. Off-policy algorithms, on the other hand, can be trained on data gathered by a different policy or, in most cases, a different iteration of the policy. This allows us to efficiently reuse exploration data by storing them in an experience replay buffer. The neural network updates are then performed using random mini-batches sampled from the buffer. This makes off-policy algorithms more data-efficient because the collected data are reused for learning; however, they tend to be less stable because data are used that have been collected by a previous policy iteration. Because off-policy algorithms can be trained with data from different policies, they enable the use of multiple robots to train one policy, as carried out by Gu et al. (2017), effectively reducing the learning time by parallelizing the exploration episodes to multiple robots and also introducing expert demonstrations into the buffer to guide the learning.

### 2.2 Combining imitation and exploration learning

Imitation learning, also known as LfD, introduces some considerations: how to demonstrate, how much to demonstrate, and the quality of demonstrations. The easiest and most intuitive way to record demonstrations for humans is to have a sensory system, such as cameras, that observes the user performing the task. However, this introduces the problem of how to translate the observed human motions into robot motions. On the other side of the spectrum, the user can demonstrate the task by teleoperating the robot. This allows the system to connect onboard sensory information with goal control actions. However, it is more difficult for the user to perform the task using a teleoperation device, and it introduces a learning curve.

There have been efforts to generate large demonstration datasets from videos of robots interacting with objects, as shown by Dasari et al. (2020). However, the recent trend appears to be toward using teleoperation and focusing on improving the teleoperation interface to make it more intuitive for the user, thereby reducing the learning curve (Zhao et al., 2023; Wu et al., 2024). Mandlekar et al. (2022) show that learning from human demonstrations can be tricky even when teleoperating. We use our own sensory system to make decisions that might not be captured by the robot’s sensory system. In addition, we can use events/observations that occurred earlier in time to make a decision at the current time, whereas the most common neural network architecture simply uses the current state to decide on what action to take next. Finally, when demonstrations are provided by multiple humans, they can introduce a large variance in both task execution proficiency and strategy to solve the task. This can even be the case for a single demonstrator as there is a significant amount of variability in the human performance of manual and teleoperated tasks.

The size and quality of the dataset can also significantly affect performance. The dataset must provide a good representation of the variance in the task, such as the variance in the position and orientation of the object of interest. The quality of the demonstrations is also important because imitation learning algorithms can at best only be as good as the given demonstrations.

As described in the previous section, the critic in actor-critic methods tends to overestimate the values of unseen state–action pairs, denoted as *extrapolation error* by Fujimoto et al. (2019). This is less of a problem in an online RL setting, where the policy can explore those areas in the state–action space and, therefore, gather data to correct the critic’s false beliefs. However, this means that modern off-policy deep RL algorithms are not truly off-policy because they only learn successfully if the data in the replay buffer are gathered by a previous iteration of the policy; that is, the dataset is correlated to the true distribution under the current policy. Therefore, using only a fixed replay buffer that contains, for example, data gathered during the training of another policy or only expert demonstrations leads to unsuccessful learning. This type of RL, in which no additional exploration is possible, is referred to as offline RL or batch RL. The idea is that using a large dataset with good, medium, and bad demonstrations, a policy can be trained that performs the best actions contained in the dataset. In contrast, BC algorithms would learn to perform the average action contained in the dataset because rewards are not taken into account in a supervised learning setting; therefore, the policy has no way of knowing what actions are good or bad.

The TD7 algorithm implementation by the original authors has both online and offline versions (Fujimoto et al., 2023). In the offline RL setting, a BC loss is added to the policy update inspired by Fujimoto and Gu (2021). However, because nothing is added to the critic loss, the learning will not be successful if the overestimation of unseen state–action pairs is too large. We will show later in the simulation experiments how the success rate of offline learning appears to be correlated with the extent to which the state–action space is covered in the dataset.

In general, a policy trained in an offline setting can only be as good as the best demonstrations, and further online fine-tuning is required to improve it. Nair et al. (2021) show that online fine-tuning after both pretraining a policy in a supervised manner and pretraining in an offline RL setting does not work. When using BC to pre-train the policy (actor), the performance degrades quickly below the level of a policy that is trained from scratch and only catches back up much later. This was also observed by Nair et al. (2018). When pretraining in an offline RL setting, the policy performance does not increase significantly after new online data are introduced. Martin et al. (2021) show that when pretraining the policy in a supervised manner for the right amount, the learning performance can improve, but pretraining too much will drastically decrease performance. Therefore, it appears to be a brittle hyperparameter to add.

We propose a simple change to the TD7 algorithm, inspired by Nair et al. (2018), where we add a second replay buffer that is used for storing demonstrations. The algorithm was run in the normal online version. At each training step, a batch is created by sampling both the online replay buffer and the demonstration replay buffer. This should guide the policy toward the high-reward regions exposed by the demonstration and also allow for exploration, resulting in a higher performance level than that demonstrated by the expert. The ratio of the amount of data sampled from each buffer is an additional hyperparameter that must be selected.

### 2.3 System architecture

A robot with passive compliance reduces the complexity of solving contact-rich tasks (Wang et al., 2019). This also extends to the use of RL to learn these types of tasks (Martin-Martin et al., 2019). An articulated soft robot has passive compliance through soft materials in the actuators, while being less complex to control and more energy-efficient than a continuous soft robot. The GH360, shown in Figure 1, is a human-inspired robotic arm that is driven by soft tendons. It was originally published as an open-source project under the name Gummi Arm (Stoelen et al., 2022). Fieldwork Robotics (Cambridge, United Kingdom) further developed it into what was called the GH2 arm. In this section, we describe the hardware of the GH360 and how it differs from the original Gummi Arm. We also describe the software developed for the robot and the setup used for the learning.

#### 2.3.1 Hardware

**FIGURE 2:**
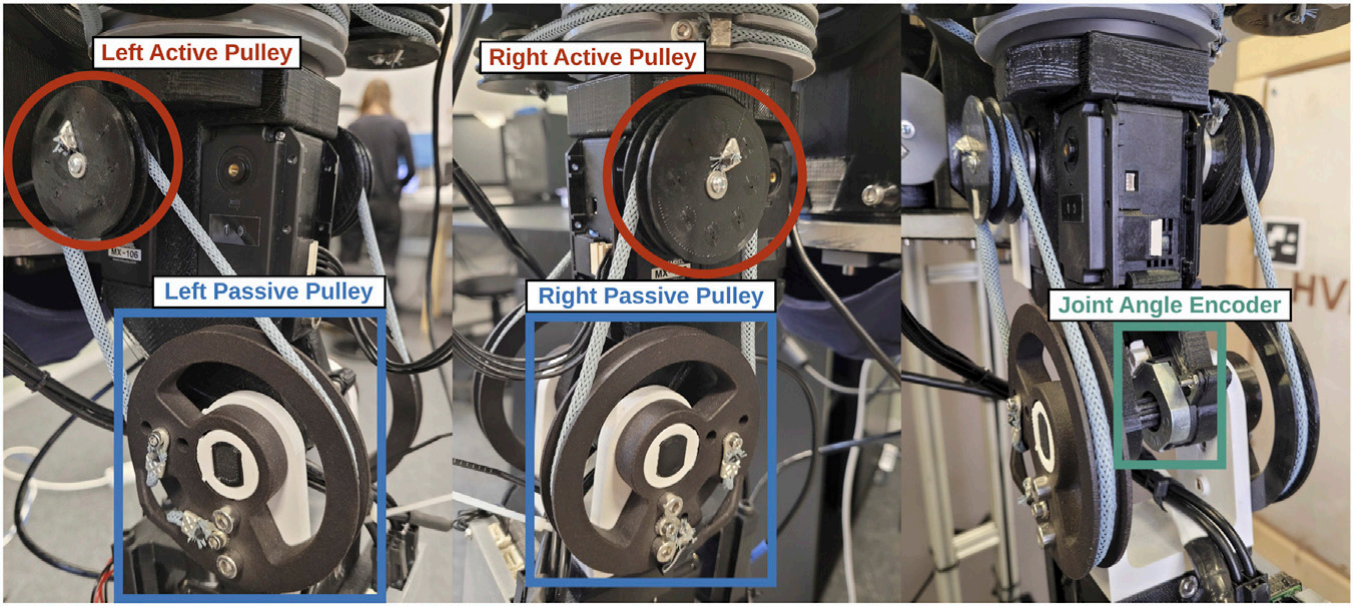
GH360 VSA joint design. Each joint has two motors in a bidirectional agonistic/antagonistic setup, allowing for stiffening of the joint by co-contracting, that is, moving the motors in opposite directions. The active pulleys are driven by the motors, whereas the passive pulleys are connected to the center of the joint and are driven by the active pulley via soft tendons that feature a nonlinear stress–strain curve. Encoders are mounted in the center of every soft joint to provide joint angle data.

The robot has seven joints, six that are driven by variable-stiffness actuators (VSAs), and one that is driven directly by a single motor. Each variable-stiffness actuator has the following components shown in Figure 2: two motors, two active pulleys, two passive pulleys, four tendons, and one rotary encoder. The two active pulleys are connected to a motor on each side of the joint. Two tendons connect the active and passive pulleys. Passive pulleys are mounted on each side of the joint center. A rotary encoder was also installed inside the joint to detect the joint angle. In total, the arm uses ten Dynamixel MX-106 motors on the shoulder and upper-arm parts of the robot and three Dynamixel MX-64 motors on the lower arm. The six joint angle sensors are CUI AMT222B-V single-turn absolute position encoders. Only six encoders are used because one joint, namely, the forearm roll, is directly driven by a motor, and the motor angle is also the joint angle. The arm structure can be 3D-printed using common materials such as acrylonitrile butadiene styrene (ABS) or polyethylene terephthalate glycol-modified (PETG). The only exceptions are the three metal plates used to mount the first joint to the base. This provides the user with the freedom to easily and quickly change the design of the arm or replace broken pieces.

The main design differences between the GummiArm and GH360 are the bidirectional agonistic/antagonistic VSA setup and tendon design. The GummiArm had only one tendon per motor connected to the joint, which limits the maximum torque of the joint to the torque of a single motor. The bidirectional setup on the GH360, on the other hand, allows both motors to pull in the same direction, essentially doubling the maximum joint torque. The original tendons on the GummiArm were built by wrapping a stiff nylon thread around a stretchable 3D printer filament. During extension, the stiff nylon starts squeezing the filament, taking over incrementally more of the load, resulting in a near-quadratic stress–strain curve. Although these tendons have the correct properties for the VSA setup, they are time-consuming to produce, making it difficult to produce multiple tendons with the same or similar stress–strain properties, and the properties change over time. This is because two tendons that behave the same must have the same spacing between each rotation of the nylon thread. Over time, when the tendons go through extension/relaxation cycles, the loops tend to bunch up, changing the stress–strain properties. Therefore, in the new composite tendon design, the inner filament is still a flexible 3D printer filament, namely, Ninjaflex. However, instead of the nylon thread, we use a braided nylon sleeve as the outer layer. More details on the evaluation of the new tendon design and how to properly set up a joint can be found in our previous study (Elstner et al., 2023).

#### 2.3.2 Software

**FIGURE 3:**
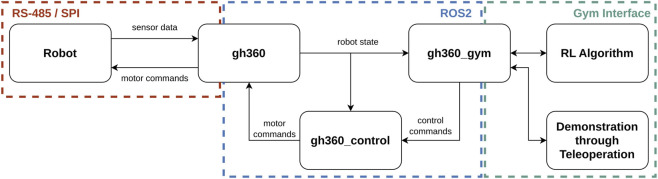
Software stack for RL on the GH360. The robot and computer communicate via serial communication. We use ROS2 as middleware between the software packages. The *gh360_gym* package provides a gym interface, which is used by RL algorithms and teleoperation devices.

Three different communication levels are used within the software architecture, as visualized in Figure 3. At the lowest level, RS-485 is used to communicate with the motors, and serial peripheral interfaces (SPIs) are used to communicate with the encoders. At the middle level, ROS2 is used to communicate between the different software packages of the robot. At the top level, a gym API is provided, which RL algorithms can use to learn with the robot. We used ROS2 for our middleware because it allows for the easy incorporation of other available software packages in the ROS2 ecosystem, such as camera drivers or fiducial marker recognition, and supports real-time communication. The main three software packages used by the robot are *gh360*, *gh360_control*, and *gh360_gym*.

The *gh360* package handles serial communication with the motors and encoders on the arm and exposes several ROS2 topics to communicate with the arm. The main functionality here is to transform data between integer values used in serial communication and float values of standard units. The package also handles any offsets or direction modifications defined in the configuration files and filters the sensor data.

While *gh360* is responsible for sending commands to the motor, *gh360_control* contains higher-level controllers, such as a joint position or end-effector (EEF) velocity controller. By default, the motors are controlled using velocity commands. The package provides the following controllers: motor position, joint position, joint velocity, and EEF velocity. Both the motor position and joint position controllers use a simple P controller, which uses the current motor position and current joint angle as feedback signals. The joint position controller also modifies the goal velocity calculation by multiplying it with the ratio between the passive and active pulleys on each joint. The joint velocity controller is an open-loop controller that calculates the goal motor velocity by multiplying the goal joint velocity with the pulley ratio. It is used in the EEF velocity controller, which translates goal EEF velocity into goal joint velocities using the Kinematics and Dynamics Library (KDL) from Orocos. The reference frame for the linear velocities is the base frame of the robot. The angular velocities are in the tool center point (TCP) frame. This choice was made through empirical testing of what was found to be the most intuitive for the authors to teleoperate the arm to provide expert demonstrations.

The custom gym environments in the *gh360_gym* package provide a standardized method for RL algorithms to control the robot. Gym environments generally consist of three main functions: *make()*, *reset()*, and *step()*. The *make()* function is used to instantiate the environment and manage the setup process. The *reset()* function is responsible for moving the robot to the initial position from which the episode is started and also must reset the rest of the environment, for example, that the door is closed. It also returns an observation list after the reset is completed to obtain the initial state of the environment. The *step()* function is used to control the robot. The function takes as an input the action the robot is supposed to take and returns the observed state of the environment, the reward if the task has been completed, and any additional information the environment provides. The action is dependent on the underlying controller, which is defined in the *make()* function. The *gh360_gym* environment sends actions to the *gh360_control* package and receives the observed state of the robot from the *gh360* package. All of this communication is achieved through ROS2.

### 2.4 Contact-rich task learning on the GH360

**FIGURE 4:**
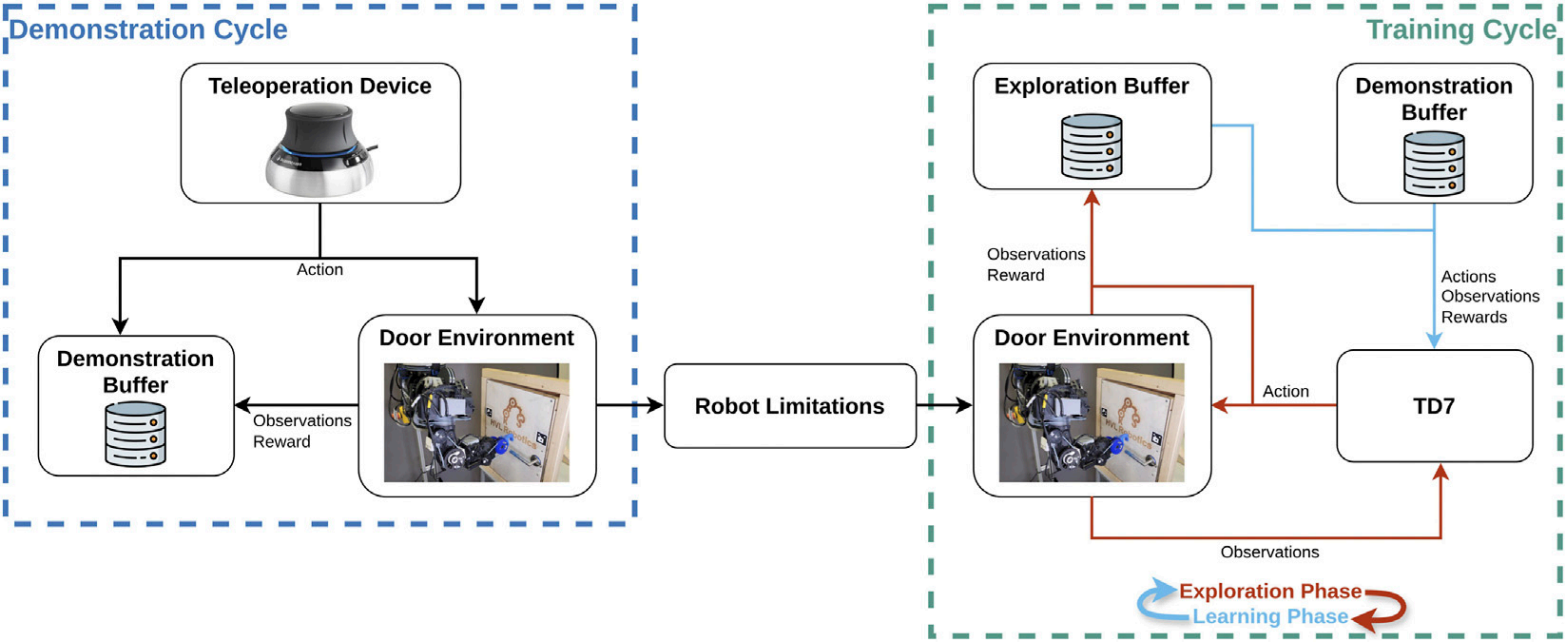
Training process for learning contact-rich tasks on the GH360. During the demonstration cycle, the user demonstrates the task to the robot through teleoperation using a SpaceMouse. Actions, observations, and rewards are stored in the demonstration buffer. Additionally, the joint and motor states are recorded and used to set limitations for the robot during the training cycle. The training cycle consists of two phases: exploration and learning. The robot starts in the exploration phase, in which the policy produces actions according to the given observations. In this phase, all actions, observations, and rewards are stored in the exploration buffer. After the exploration phase, the robot transitions into the learning phase, in which a batch consisting of data from both the exploration and demonstration buffers is used to train the TD7 policy. Subsequently, the robot transitions back into the exploration phase. This cycle is repeated for a given number of exploration episodes.

Combining the previously discussed concepts, we use the following process, as shown in Figure 4 to learn contact-rich tasks using the GH360. Initially, the user demonstrates the task to the robot, which we call the demonstration cycle. Subsequently, the robot transitions into the training cycle, in which it cycles between the exploration and learning phases. In the exploration phase, the DRL policy controls the robot and produces actions given observations. In the learning phase, the policy is trained using a batch of data sampled from both the exploration and demonstration buffers.

The demonstrations are performed through teleoperation using a SpaceMouse, which is a 6-DoF joystick. The SpaceMouse output is sent to the EEF velocity controller. We use an EEF controller because Martin-Martin et al. (2019) showed that DRL algorithms perform better with controllers in EEF space than controllers in joint space. We use a velocity controller because it ensures smooth movements throughout the episode. Because gym environments use a *step()* function that is executed at a specific frequency, if a position controller is used and the position is reached before the next time step, the motors would stop and start, leading to jittery movements. Because the EEF velocity controller is open-loop and there are soft tendons between the joints and the motor, the resulting motion of the TCP will not be exact, but rather, the user and later the RL policy must correct these inaccuracies. However, a closed-loop controller would fight against the inherent compliance of the tendons, making the use of soft materials self-contradictory (Santina et al., 2017).

The demonstrations are performed by sending SpaceMouse commands as actions to the gym environment, as shown in Figure 3. This ensures that if the robot sends the same action in the same state, it will produce the same output. Teleoperating through the gym environment means that no post-processing must be performed on the data. Actions, observations, and rewards during the demonstration cycle are stored in a demonstration buffer. Additionally, all motor and joint states are recorded. After the demonstration cycle is completed, the maximum and minimum joint angles and motor currents recorded during the demonstrations are used to limit the robot during the training cycle.

The training cycle begins with an exploration phase, in which the current iteration of the policy controls the robot, sending actions given observations. The exploration phase lasts one episode, during which all actions, observations, and rewards are stored in the exploration buffer. After the exploration phase, the robot transitions into the learning phase, during which a batch of data is sampled from both the exploration and demonstration buffers and used to train the different neural networks of the TD7 algorithm. This is repeated for the same number of iterations as time steps taken during the exploration phase. Subsequently, the robot transitions back into the exploration phase. This cycle is repeated for a specified number of exploration episodes. Martin-Martin et al. (2019) showed that using a Cartesian space as an action space increases the performance of DRL algorithms. Therefore, the output of the policy is EEF velocities. However, because of the inaccuracies of the soft materials and open-loop controller, the policy must also learn to correct these, as described earlier.

Performing RL on a real robotic system, instead of a simulation, introduces some challenges (Ibarz et al., 2021). The main problems are described as robot persistence and safe learning. As previously discussed, RL requires a large amount of data for successful learning. When gathering these data on a real system, robot persistence, that is, the capability of the robot to collect data and train with minimal human intervention, becomes more challenging.

First, resetting both the robot and the environment is trivial in simulation but often requires a special experimental setup and manual engineering when learning on a real robot. For example, when learning to open a door, the experimental setup must be designed to allow the door to close automatically using, for example, a spring or motor. In addition, resetting the robot itself can require intricate reset trajectories in situations such as when the robot EEF becomes entangled with the door handle.

Second, the robot itself must be able to explore the environment without causing damage to itself or the environment, which would require human intervention to repair or replace what is broken. In other words, the robot must be able to explore safely, that is, safe learning. This can be achieved by restricting the action space to allow only safe actions. Restrictions can include limiting the EEF pose to a defined area, constraining velocities, or using force-torque sensing to stop the robot on impact. In contact-rich tasks that require the robot to apply forces to the environment to complete the task, simple stopping on collision will not work. Instead, the allowed forces must be restrained to an amount that is sufficient to complete the task.

Our proposed algorithm uses expert demonstrations to define the limits for joint angles and motor torques during exploration. During exploration, random noise is added to the actions chosen by the policy. This can lead to jerky motions, which may damage the gearbox and actuator. There have been many different approaches to smoothing actions, such as using a low-pass filter or penalizing jerkiness through the reward function. However, this is not a significant problem for the GH360 because it has soft tendons between the motors and joints that passively dampen any sudden motor movements.

In general, it is important to recognize unsafe situations and have a way to recover from them or shut down the robot completely. This can be achieved through heuristic-based approaches, such as collision checking or torque monitoring. It is more difficult in contact-rich tasks to find limits that do not inhibit the robot from learning the task and would therefore require careful manual task-specific tuning. Another approach to increase safety is to penalize unsafe behaviors using the reward function. However, this means that the robot must perform that unsafe action at least once to learn from it. In addition, careful tuning is required because excessive penalization could result in the robot not wanting to take any actions. This could be a good approach if learning is performed in simulation and only the trained policy is executed on the real robot. The passive compliance of the GH360, combined with limits on the allowed motor velocities, reduces the number of unsafe situations. What is left is covered by limiting the allowed motor currents to an amount that is sufficient to solve the task. Because we infer those limits from the given demonstration, no manual tuning is required.

## 3 Evaluation setup

**FIGURE 5:**
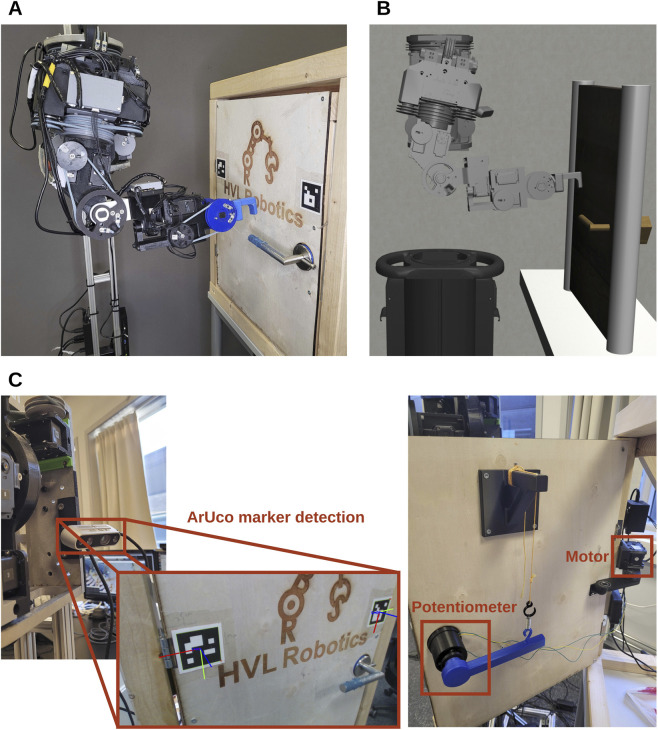
Learning environments. **(A)** Real setup, **(B)** simulation setup, and **(C)** sensor setup for the real environment. A camera is used to detect the ArUCo markers on the door to determine the position and orientation of the door. For the position of the door handle, we used a static transformation from the door pose. A potentiometer was mounted at the back of the door to determine the door handle angle. A motor is connected to the back side of the door through a 2-DoF arm with passive joints. The angular motor position is used to determine the hinge angle of the door and to automatically close it during the resetting phase.

The proposed methodology was evaluated on the problem of opening a door, shown in Figure 5. The task consists of three phases. First, a free-space movement during which the robot must place the hook on the door handle, ideally at a position that is as far away from the handle joint as possible so that less force is required to push down the handle, but not so far that the hook will slip off when force is applied. Second, the robot must apply force onto the handle to push against the spring that will return the handle to its initial position if it is released. The direction of force application is important because an incorrect direction can result in the EEF slipping off the handle. Third, the robot must open the door while continuing to apply force on the handle (at least in the beginning) and is kinematically constrained by the one DoF of the door.

The expert demonstration dataset was acquired by teleoperating the simulated robot using a SpaceMouse. The demonstration is performed via the gym environment; that is, the commands from the SpaceMouse are used as actions in the *step()* function, which allows for recording data in the format that is used in off-policy RL buffers: observation, action, next observation, and reward.

### 3.1 Door-opening task

The reward function of the door-opening task was divided into four phases: reaching for the door handle, pushing down the handle, pulling the door handle to open the door, and releasing the handle once the door is opened. The task is successful, and a full reward of 1 is given at each timestep if the hinge angle is greater than 0.4 rad (meaning that the door is open), and the handle angle is smaller than 0.1 rad (meaning that the handle is released). Otherwise, the reward is as follows:

- Pulling open the door: If the hinge angle is bigger than 0.1 rad, the reward is given by Equation 1:

$$r=0.5+0.25*\left|\frac{hinge\text{\_}q_{c}}{hinge\text{\_}q_{g}}\right|,$$ (1)

where $hinge\text{\_}q_{c}$ is the current hinge angle and $hinge\text{\_}q_{g}$ is the goal hinge angle.

- Pushing down the handle: Else if the handle angle is bigger than 0.1 rad, Equation 2 gives the reward as

$$r=0.25+0.25*\left|\frac{handle\text{\_}q_{c}}{handle\text{\_}q_{g}}\right|,$$ (2)

where c and g are the current and goal handle angles, respectively.

- Reaching for the handle: Else, the reward is given by Equation 3:

$$r=0.25*\left(1-tanh\left(10*d\right)\right),$$ (3)

where d is the distance between the tool center point (TCP) and a predefined point on the door handle.

The reset position of the robot was defined as the following joint angle configuration: [0.02, −0.19, 0.15, 1.58, 1.87, 0.0, 0.0]. The evaluation criteria used in the different experiments were as follows:

- Sample efficiency: We use the reward value over time to evaluate sample efficiency with a specific interest in the performance towards the end of the experiment run.
- Reliability: We use the mean reward and variance over the whole learning run to determine the reliability of the approach. A higher mean reward indicates that the policy spent more time exploring high-reward regions, which means that the robot performed actions closer to the desired behavior and fewer unexpected/unwanted movements. A lower mean variance indicates that the algorithm has a more stable performance from run to run.
- Task completion and final performance: We run 10 evaluation episodes after training to determine task completion rate and performance in terms of the mean reward.

### 3.2 Evaluation in simulation

To perform an initial evaluation of the additional hyperparameters introduced by our methodology and compare the different variations of the TD7 algorithm, we used a simulation environment because it runs faster than real time and allows us to run experiments in parallel. The simulation was performed in MuJoCo (Todorov et al., 2012) using the robosuite framework (Zhu et al., 2025), which comes with a door-opening task implementation. We adapted the task to match the real setup in terms of the position and orientation of the door, the handle angle required to open the door, the dimensions of the door handle, and the reward system.

We also extended the framework by including the GH360. The robot used for the simulation experiments did not use tendons but rather had an actuator driving each joint directly. This makes the simulation more stable and faster, and it should not significantly affect which hyperparameters or learning algorithms perform best. The simulation setup is shown in Figure 5B. The simulated robot was controlled using an operational space fixed impedance controller at a control frequency of 20 Hz. Each episode had a fixed length of 500 timesteps. The joint angles for each reset were varied using Gaussian noise with a magnitude of 0.02. The position of the door was varied by ± 10 mm in x and y , and the orientation varied by ± 0.17 rad around z . The following algorithms were evaluated:

- TD7: The online variant of the TD7, which is an RL algorithm without demonstrations. We used the implementation from Fujimoto et al. (2023).
- TD7 Offline: The offline variant of the TD7. We used the default implementation of the original authors but evaluated it with a different type of dataset than that used in Fujimoto et al. (2023).
- TD7+Demo: Our proposed approach.

Because safety was not an issue in the simulation, no joint angle or torque constraints were applied to the robot after gathering the demonstration data.

#### 3.2.1 Ablation study

Our proposed TD7+Demo algorithm introduces two additional hyperparameters: the number of demonstration episodes and the demonstration-to-exploration ratio in each learning batch. The percentage of demonstration data in each learning batch was set to either 10% or 50% in previous studies (Reddy et al., 2020; Nair et al., 2018). Therefore, we conducted learning runs with 10%, 20%, 50%, and 75%. The number of expert demonstrations used during learning can be difficult to determine because it depends on the variation in the task. It is desirable to have enough demonstrations to cover the variations between task executions. However, at a certain point, additional demonstrations do not add valuable information, and providing too many demonstrations can be tedious for the operator. In this study, we evaluated using 5, 10, 20, and 50 demonstrations in the demonstration buffer.

#### 3.2.2 Offline learning

The TD7 algorithm implementation by the original authors included the possibility of training a policy offline using a demonstration dataset. As discussed in Section 2.2, offline RL promises to learn the best actions available in a dataset that contains good, medium, and bad demonstrations. However, the evaluation of the offline variant of the TD7 algorithm performed by Fujimoto et al. (2023) uses very specific datasets. The datasets used were the medium, medium-replay, and medium-expert from the D4RL datasets (Fu et al., 2020). All of these use a partially trained policy; that is, the learning was stopped early to gather data. In the medium dataset, the partially trained policy was used to gather demonstrations. The medium-replay dataset consists of all data gathered to partially train the policy, that is, the buffer data. The medium-expert dataset mixes an equal amount of data gathered from a partially and fully trained policy. This results in a good representation of the overall function distribution, but creating these types of datasets is not realistic in a real-world scenario because another policy must be trained first to gather the data. Therefore, we created datasets that resembled a similar structure but used a combination of human demonstrations and random movements:

- Expert: successful demonstrations by an expert user teleoperating the robot using the SpaceMouse.
- Random Expert: combines the expert dataset with a random dataset from which a random action is chosen at each time step for the same number of episodes as the expert demonstrations.
- Gradual Random Expert: In each episode, we choose at random one of the expert demonstrations and replay that demonstration for a random number of time steps. In the remaining time steps of the episode, we used random actions. The number of episodes gathered is equal to the number of expert demonstration episodes. The data were combined with the random expert dataset to produce the Gradual Random Expert dataset.

#### 3.2.3 Final evaluation

Finally, we compare the different TD7 algorithm variations against each other over 10,000 exploration episodes to determine the performance given ample time and more than 200 exploration episodes, as this is a more reasonable timeframe to execute on a real system. It should be noted that the offline variant of the TD7 does not actually gather additional exploration episodes but has 10 training cycles between every evaluation cycle, which is therefore equivalent to the online variant.

### 3.3 Evaluation in the real world

When applying RL to real robots, additional challenges emerge that are less of an issue in simulations. Specifically, it takes more time to run the same number of timesteps, perfect information is not available (meaning that data needed for observations or the reward function requires an actual sensory system), and automatic resetting of the environment without requiring human intervention is a non-trivial challenge. To reduce the time required for the experiments, the episode length was reduced to a value that was slightly longer than the slowest recorded expert demonstration. The number of episodes per learning run was also reduced to an approximate number that produced stable results in the best-performing approach in simulation.

The door, shown in Figure 5C, is self-built but contains an off-the-shelf door handle and is equipped with sensors and markers that are a reliable source of data needed for the reward system. The front panel has two ArUCo markers that are used to detect the position and orientation of the door through a camera mounted to the base of the robot. We used static transformations to obtain two handle positions and used the mean to calculate the reaching reward. The handle angle is determined using a potentiometer attached to the back of the door. A motor is mounted on the door frame and connected through two passive joints to the door back panel. It is used to obtain the hinge angle for the reward system and automatically close the door if it is opened during an episode.

**FIGURE 6:**
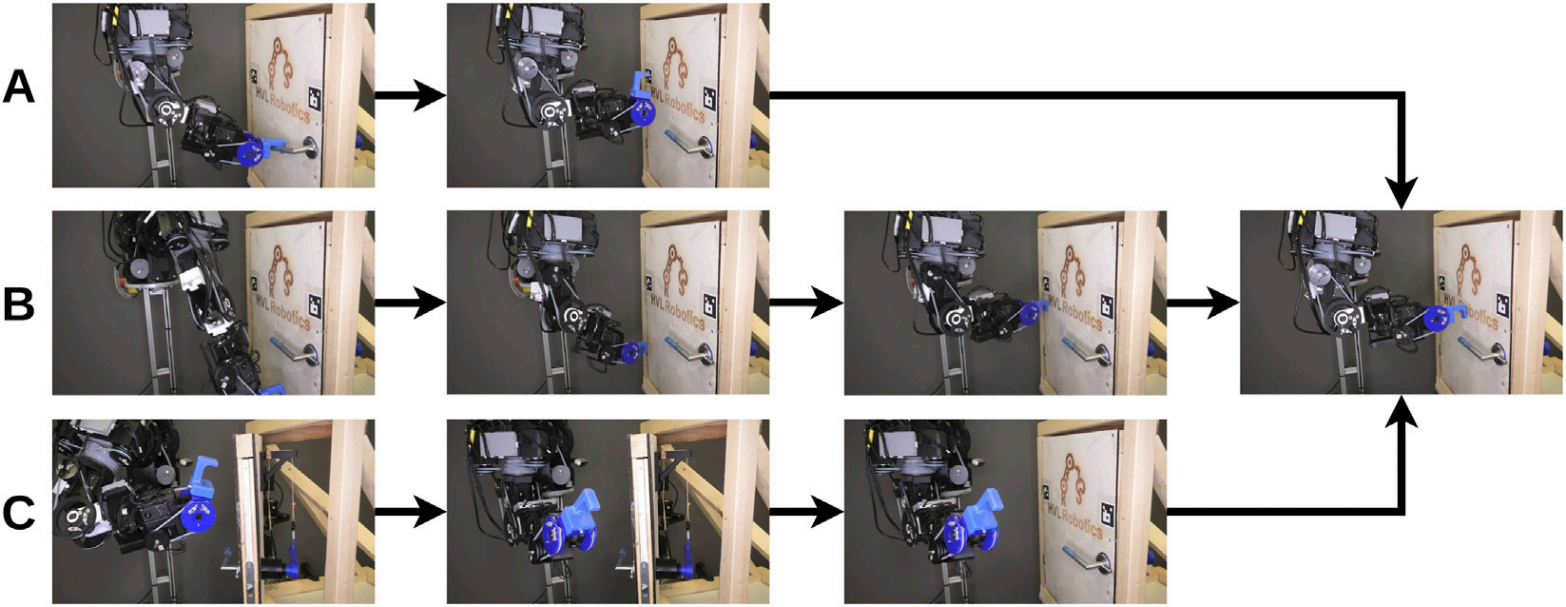
We use three different reset behaviors for the door-opening environment. The execution logic is as follows. **(A)** The TCP is above the door handle and the door is closed. **(B)** The TCP is below the door handle and the door is closed. **(C)** The door is open or partially open.

Because of the passive compliance of the robot, a sophisticated path planner is not required to reset the environment. Instead, we simply define joint configurations to which the robot moves. Specifically, we considered three different scenarios: the door is open, the door is closed, and the EEF of the robot is under the door handle, and the door is closed with the EEF above the door handle. The different scenarios are shown in Figure 6.

- The door is open: The robot is moved into a configuration that does not block the door. This is because the door potentially needs to be opened more for the motor to build sufficient speed to overcome the spring-loaded closing locking mechanism of the door. After the door is closed, the robot moves to the reset position above the door handle. A visualization of this behavior is shown in Figure 6C.
- The door is closed: If the EEF of the robot is above the door handle, then the robot will move into the reset configuration but with a high wrist pitch angle, as visualized in Figure 6A, to ensure that the robot is untangled from the door handle before moving into the actual reset position. If the EEF is below the door handle, the robot moves through two other configurations first, as visualized in Figure 6B, before moving to the reset position to avoid collisions with the door handle.

Additionally, a safety system is in place that stops the learning and waits for human intervention if the robot exceeds the allowed motor currents or if the reset fails 10 times. The user can then restart/reset the system and continue learning using our modified TD7 implementation, allowing for the stopping and continuation of a learning run.

The following approaches were compared with each other for the real door-opening task:

- Demonstration replay is the only approach that does not use any learning. We randomly selected one of the recorded demonstrations and replayed it.
- Behavioral cloning (BC): We train a policy only on the demonstration dataset using supervised learning. The BC policy is a multilayer perceptron (MLP) with two fully connected layers, each with 256 neurons. The policy is trained on the dataset until the maximum mean squared error loss in the last 500 training iterations is below 0.01.
- TD7: This represents learning without any demonstrations using only RL. This is the original implementation from the authors of the algorithm, with only a small modification, as described earlier. The hyperparameters of the TD7 algorithm were the default values from Fujimoto et al. (2023).
- TD7 + Demos: Our proposed approach, in which we use a combination of exploration and imitation. The robot is trained on the demonstration dataset and can also gather additional data through exploration. The TD7 algorithm was used for exploration, as described in the previous point.

To investigate the task completion rate and final performance of the system, 10 evaluation runs were performed at the end of the learning process. To expose the robot to additional variance introduced by the flexible tendons, a specialized reset behavior was used for these episodes. Ten random combinations of the min and max positions for each joint were generated. After each normal reset of the system, the robot moved into one of these positions before moving back to the normal reset position used during training. Because we are using a joint position controller, the joint angles at the start of each episode will be fairly similar, but the motor positions will vary. The maximum and minimum joint angles were determined according to the robot limits generated from the demonstrations. Although the combinations were initially generated randomly, we used the same list for every experiment to ensure a fair comparison. The list of combinations is provided in the Supplementary Material.

## 4 Results

The proposed methodology was evaluated both in simulations and on the real GH360 robot. The evaluation was performed on a door-opening task that is rich in contact forces and kinematically constrains the robot once it is in contact with the door handle. In both the simulation and real-world experiments, we first gathered a demonstration dataset using a SpaceMouse to teleoperate the robot. We used this dataset for all experiments.

### 4.1 Simulation

To generate a demonstration dataset for the simulated GH360, a SpaceMouse (3Dconnexion, Germany) was used to record 50 successful door-opening episodes. Each episode is set to have a fixed episode length of 500 timesteps, as empirical testing showed that this amount of time is sufficient to reliably demonstrate the task successfully. To evaluate how the newly introduced hyperparameters in our proposed approach influence performance, a mini-ablation study was performed on the number of demonstration episodes in the demonstration buffer and the percentage of demonstration data in each learning batch. Each experiment was repeated five times to indicate the reliability of the performance. Because randomness is introduced during exploration episodes, 10 evaluation episodes are run every 10 exploration episodes to evaluate the performance at different points in time during learning. During each evaluation episode, the current policy iteration is executed without randomness. Each learning run was limited to 200 exploration episodes.

#### 4.1.1 Ablation study

**FIGURE 7:**
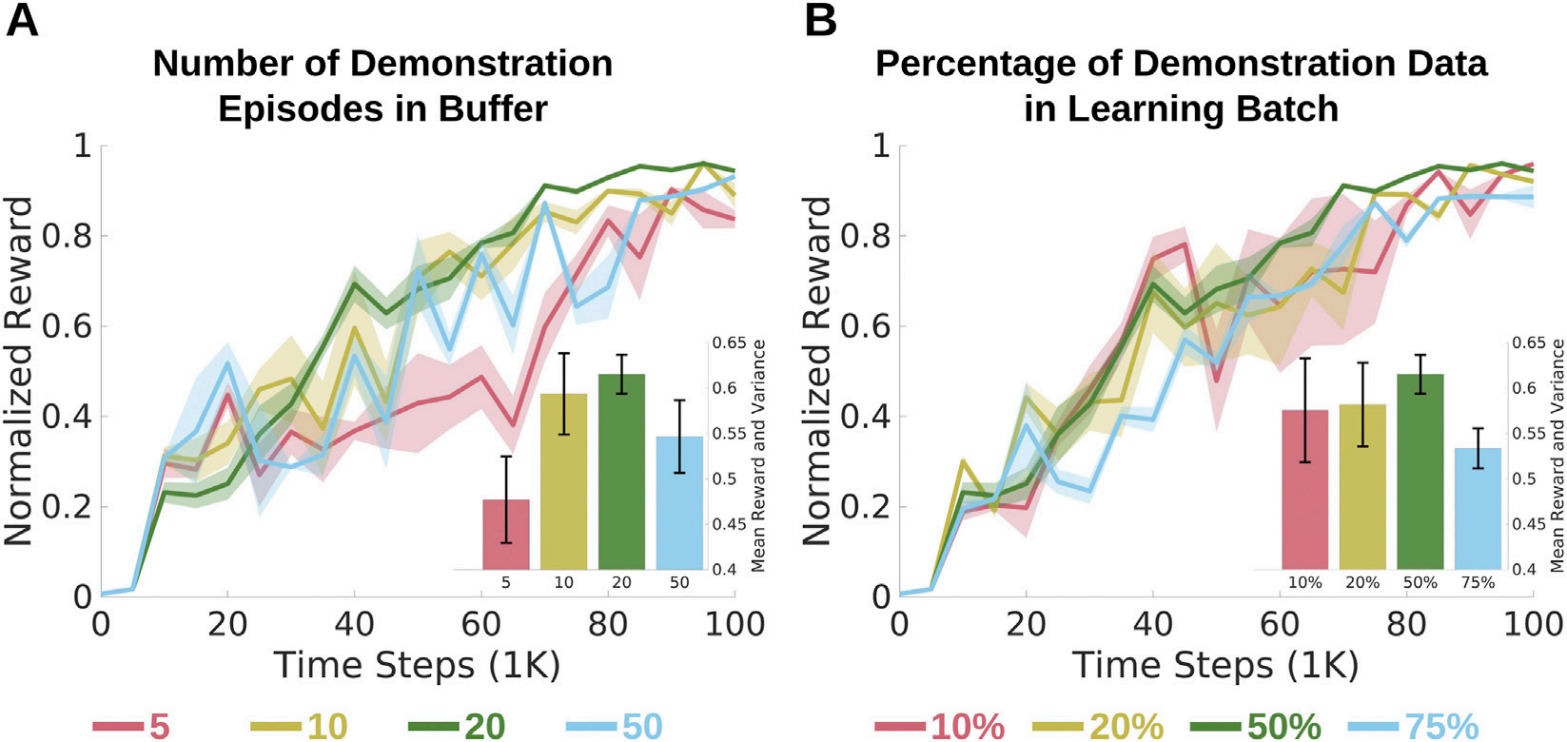
Ablation study results on the hyperparameter introduced in the TD7+Demos algorithm. Five learning runs were conducted. After every 10 exploration episodes, we performed 10 evaluation episodes. The lines in the graph represent the mean, and the shaded areas represent the variance. The bar graph shows the mean reward and variance over all time steps. **(A)** Comparing the number of demonstrations in the demonstration buffer. **(B)** Comparing different batch ratios.

To evaluate the influence of the number of demonstrations in the buffer, learning runs were performed using the first 5, 10, 20, and 50 episodes of the demonstration dataset. For this experiment, each learning batch consisted of 50% demonstration data and 50% exploration data. The results are shown in Figure 7A. The mean of the 10 evaluation episodes was computed to calculate the performance at different time steps. The solid line represents the mean over the five learning runs, and the shaded area around the line represents the variation. The bar plot in the bottom right corner shows the overall mean reward, and the error bars show the overall mean variance. All four evaluated versions achieved a reward of more than 0.8 at the end of learning, both when using 5 and when using 50 demonstrations. However, the curves during learning appeared to be less stable and lower performing than 10 and 20 demonstration episodes. This is also reflected in the overall means and variances. Here, 20 demonstrations show both the highest reward and the lowest variance of the four possibilities.

The second hyperparameter added by the proposed approach is the ratio between the demonstration and exploration data in each learning batch. To evaluate how this parameter influences the performance, experiments were performed with 10%, 20%, 50%, and 75% demonstrations in each learning batch. For this experiment, the first 20 of the 50 demonstration episodes were used. We used 20 episodes because that number performed best in the previous experiment. The results are shown in Figure 7B. Similar to the number of demonstrations, for the demonstration percentage in each learning batch, all variations tested showed a high reward of more than 0.8 at the end of the learning run. When looking at the overall mean reward and variance in the bar plot, 50% shows both the highest reward and lowest variance of the tested approaches.

The results indicate that the performance of the proposed approach is stable with respect to the two hyperparameters it introduces because all variations tested show high performance in terms of the reward at the end of the learning run.

#### 4.1.2 Offline learning

**FIGURE 8:**
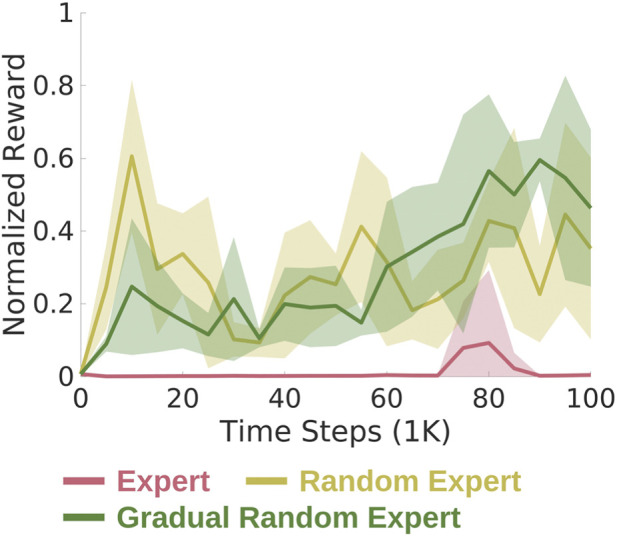
Comparing the TD7 Offline learning algorithm on different datasets. The Expert dataset consists of 50 expert demonstration episodes. Random Expert combines the Expert dataset with 50 episodes of random actions for a total of 100 episodes. Gradual Random Expert combines the Random Expert dataset with 50 episodes. During each episode, a random expert demonstration is replayed for a random number of timesteps, after which random actions are taken for the rest of the episode.

The TD7 Offline variant was evaluated on different datasets that were generated from the demonstration dataset, as described in Section 3.2.2. Each experiment was run for 200 learning training cycles. Every 10 training cycles, the current policy iteration was tested on 10 evaluation episodes. All experiments were repeated five times, and the results are shown in Figure 8. The solid line represents the mean, and the shaded area represents the standard deviation. The results indicate that using only expert demonstrations resulted in a close-to-zero performance in terms of rewards. Both the Random Expert and Gradual Random Expert performed better than the Expert dataset, with the Gradual Random Expert dataset achieving a slightly higher reward at the end of the learning run. All three approaches achieved a low final reward of less than 0.5, which means that none of them were capable of opening the door.

#### 4.1.3 Final evaluation

**FIGURE 9:**
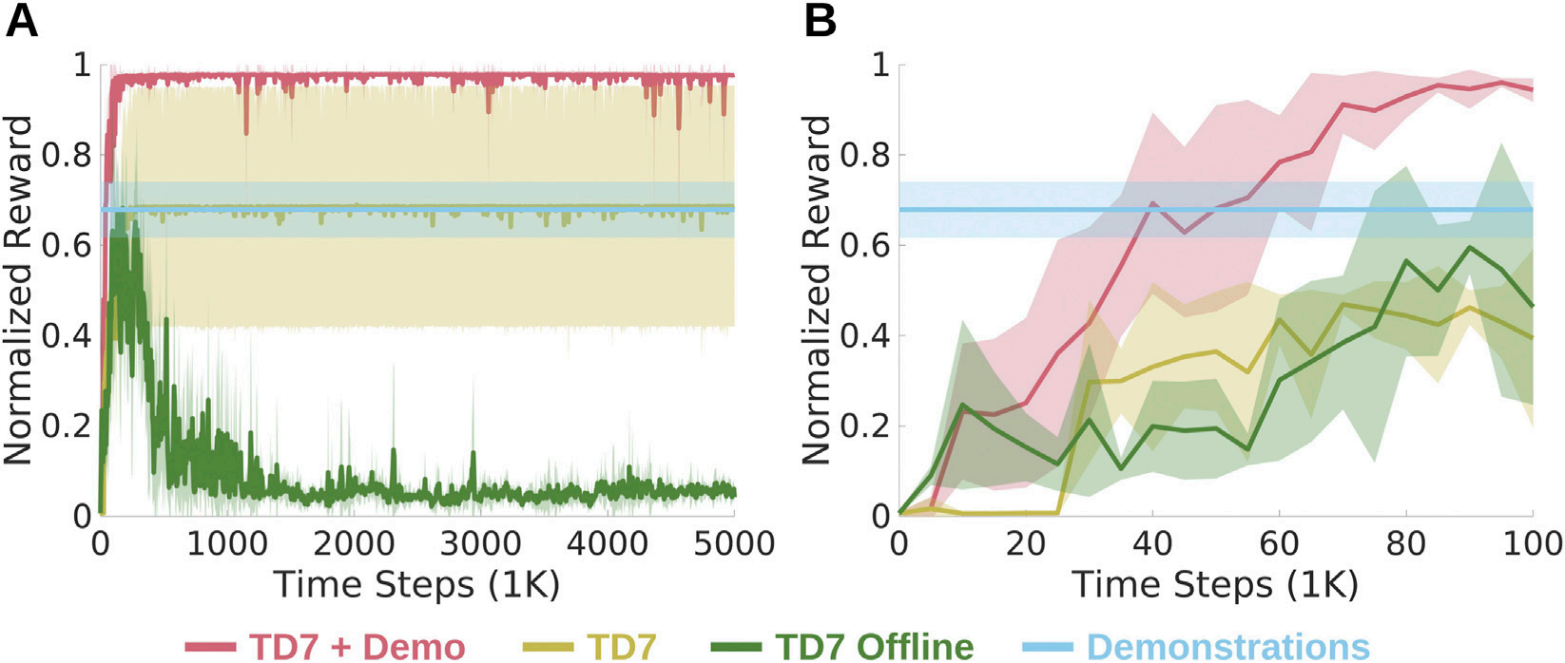
Comparing learning rates of different algorithms in simulation. TD7 and TD7 Offline are the online and offline RL implementations, respectively, from the original authors. TD7+Demo is our proposed algorithm. **(A)** Full learning length of 10,000 episodes. **(B)** First 200 episodes.

The different variations of the TD7 algorithm are compared against each other both in a long-horizon setting of 10,000 exploration episodes/training cycles and a shorter horizon of 200 exploration episodes. Each variation had five learning runs with different random seeds. The results in Figure 9A shows that the proposed approach, namely, TD7+Demo, achieves a high reward of approximately 0.97 very early in the learning run. The standard TD7 algorithm had an average reward of approximately 0.68. The standard deviation shows that the algorithm can reach a high reward comparable to TD7+Demo but can also remain stuck at approximately 0.5. The results in Figure 9B show that TD7+Demo is not only more stable but also reaches a high performance level significantly faster. The offline variant of the TD7 shows some promise early on, performing similarly to the online variant but returning to almost zero after approximately 1.5 M time steps (or 3000 training cycles).

### 4.2 Real system

To evaluate our algorithm on a real robot, we performed five learning runs of 200 episodes each. Two hundred episodes were chosen because learning/exploring on the real robot is very time-consuming, and the simulation results indicate that it is sufficient time for the TD7+Demo algorithm to reliably learn a successful policy. One episode is 130 time steps long with a step frequency of 5 Hz. The episode length was chosen using empirical testing of how many time steps were sufficient to reliably demonstrate the task successfully. To evaluate the performance during learning, the policy was tested on five evaluation episodes every ten exploration episodes. During these evaluation episodes, no exploration noise was added to the actions from the policy. The TD7 hyperparameters were the default values from the authors’ implementation. The TD7+Demo hyperparameters were set to 20 demonstrations and 50% demonstration percentage in each learning batch, as these values performed best during the simulation experiments. Twenty expert demonstrations were gathered through teleoperation using the SpaceMouse, which took approximately 17 min of wall clock time. The robot limitations generated from the demonstrations are provided in the Supplementary Material. These limitations are used during learning with both TD7+Demo and the normal TD7 algorithm.

**FIGURE 10:**
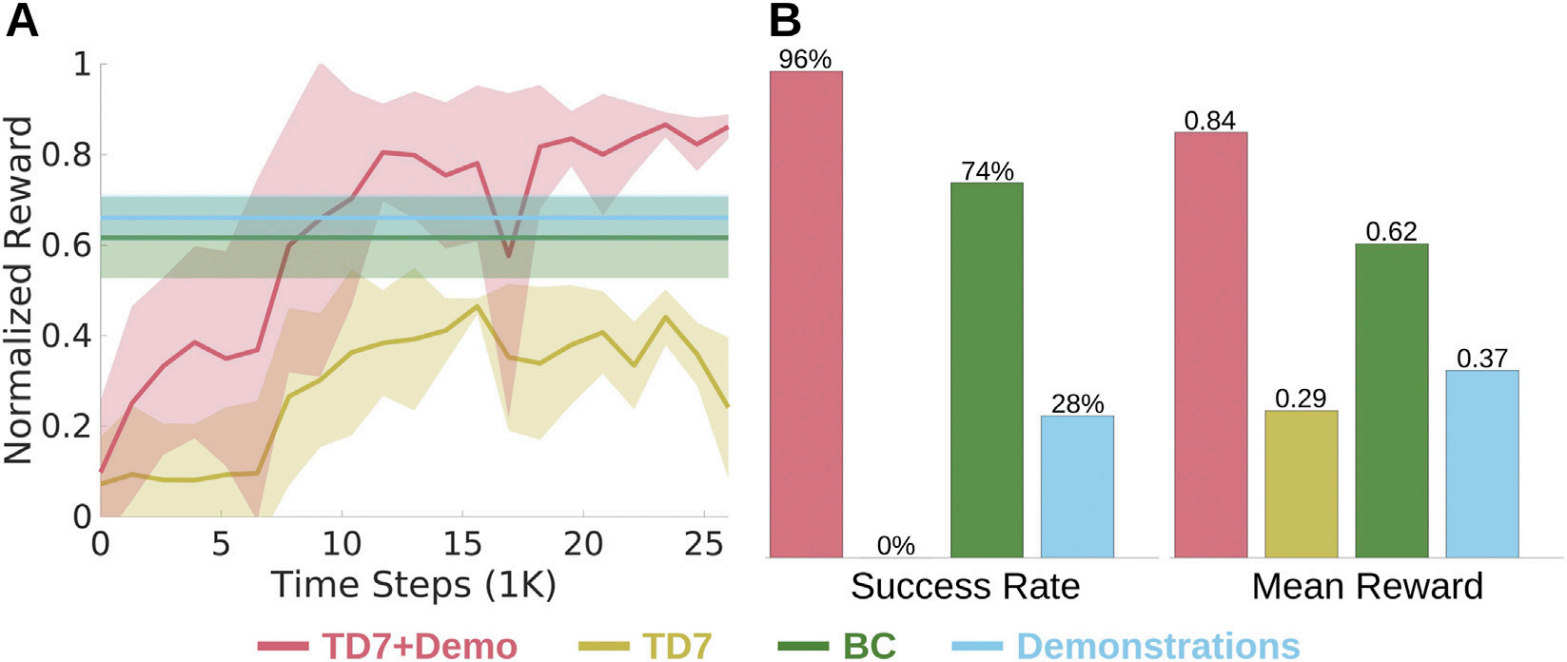
Comparing **(A)** learning rates and **(B)** final evaluation performance of different learning approaches on the real robotic system. TD7 is an online RL algorithm trained without any demonstration; BC is an MLP trained in a supervised manner on the expert demonstrations; Demonstrations in **(A)** represent the reward reached while giving the demonstrations, whereas in **(B)**, the demonstrations represent randomly replaying the recorded demonstrations for the final evaluation.

We evaluated different approaches to learning on the real GH360 robot. The TD7 algorithm represents learning from exploration, the BC algorithm represents learning from imitation, and TD7+Demo is a combination of learning from imitation and exploration. We also examined how the given expert demonstrations performed without any learning involved. Each learning approach was repeated five times. The results of the learning runs, shown in Figure 10A, appear to be similar to the results of the simulation, in which the TD7 algorithm stays under a 0.5 reward in the first 200 episodes, and the TD7+Demo reliably reaches a high reward of around 0.85. The Demonstrations line represents the mean and standard deviation of the reward achieved while providing the 20 demonstrations. The results indicate that combining imitation with exploration allows the TD7+Demo policy to outperform the expert demonstrations. The BC line represents the mean and standard deviation of the evaluation episodes after training. The BC policies can be brittle to hyperparameters, but the results indicate that it approaches the theoretical maximum, which is the Demonstrations line, because it is only trained on the expert demonstration dataset.

The results of the evaluation after training are presented in Figure 10B. TD7+Demo achieved the highest success rate of opening the door at 96%. The BC policy also achieves a relatively high success rate of 74%, but TD7+Demo reaches a higher success rate and a higher mean reward. The TD7 algorithm achieved a 0% success rate in opening the door after training, with a mean reward of 0.29. Replaying a random demonstration from the expert demonstration dataset resulted in a similarly low mean reward of 0.37 but could sometimes lead to successful door opening with a success rate of 28%.

## 5 Discussion

In this study, we evaluated how introducing demonstrations into an off-policy DRL algorithm can significantly improve the learning of contact-rich tasks on a real articulated soft robotic arm. However, some general limitations that come with performing RL instead of only performing imitation learning on a real robot still apply. First, we performed RL with shaped rewards, meaning that the policy gradually receives more rewards the closer it gets to solving the task, instead of the only reward being the full reward when the task was successful. This means that the reward function must be defined carefully to lead to a successful outcome and incentivize desired behaviors. Additionally, this implies that more data on the state of the environment are required. Instead of only detecting whether the door is open, the reward system requires data on the position of the door in relation to the robot, door handle angle, and hinge angle. We built sensors into the custom door for data reliability, but all the required information could be reasonably obtained using a computer vision approach. We chose to use reward shaping because it gives the original TD7 algorithm, which learns only from exploration, the best chance to succeed. The success reward would require the robot to randomly move in such a way that opens the door in a reasonable amount of time, which is unlikely.

Second, online rollouts on a real robot during learning require a well-designed safety system or a robot that can make mistakes without damaging itself or its surrounding environment. This is an even bigger problem when the task requires the robot to interact and apply force to objects, as it means that the torques in the motors cannot simply be limited to very low values. Because the GH360 has inherent safety properties through the soft tendons between the motors and joints, which can absorb unforeseen impacts, it was sufficient to use a limited safety system in this case. The only limitations used during the learning episodes were the minimum and maximum joint angles and motor currents observed during expert demonstrations. With this, robot learning was run in a 24/7 setup without continuous human supervision. If a motor went into a protective stop or the automatic reset failed 10 times, the learning was paused, and human intervention was required. Throughout the 2000 exploration episodes performed during the experiments (1000 with the TD7 algorithm and 1000 with the TD7+Demo algorithm), the system required seven human interventions. Five of these were with the TD7 algorithm when learning without any demonstrations, indicating that exploring close to the intended task execution is safer for the robot and its environment. This would make the TD7+Demo algorithm more robust, perform better, and be safer. However, the data size is too small to draw a clear conclusion, and it is not clear how many intervention scenarios occurred during the exploration episode or during the environment resetting. An easy improvement to the safety system could be to add a limitation to the EEF position by, for example, generating a 3D volume from the recorded EEF position data during the demonstrations and using it as an additional constraint on the allowed actions.

Third, resetting the real robot and environment is a non-trivial problem. Resetting the robot can be difficult depending on the state of the robot at the end of the episode. For example, the hook can be entangled with the door handle in a way that is difficult to undo. For a normal robot, this requires a sophisticated path planner. Using the GH360’s inherent softness, we realized that we could use three simple reset strategies, as shown in Figure 6. If the robot does not reach any of the reset configurations within 10 s, the motors are put in passive mode, that is, no current is applied to the motors, for 3 seconds. Automatic resetting of the environment, in our case the door, is important because the average learning time for each learning run was approximately 3.82 h (2.51 h without the evaluation episodes), and it would be tedious for a human operator to wait next to it to close the door manually every time it was opened. Therefore, we used a motor on the door to do this. Overall, throughout the experiments, the robot and door were operated autonomously with limited human supervision/intervention using a comparably simple safety system. While there were some minor signs of wear and tear on the door, no components of the robot had to be repaired or replaced.

According to the current evidence, the authors believe that the learning approach presented in this study applies to other contact-rich tasks. However, transferability could be limited by the manual engineering steps highlighted earlier when resetting the environment. For example, in the task of wiping spilled liquid from a surface, it would not be difficult to specify a reward function dependent on how much of the dirty surface has been cleaned, and detection could be reasonably performed using the existing onboard camera. The safety system could be used as is without any adjustments; however, resetting the environment would require an automatic spilling mechanism or regular human intervention to add new spills. The proposed algorithm is also likely transferable to other articulated soft robotics platforms, as long as an EEF velocity controller is available.

### 5.1 Demonstrations

Gathering expert demonstrations using the SpaceMouse has its pros and cons. Using teleoperation for gathering demonstrations was deemed to be the best choice for a demonstration interface in this case. Using recordings of humans opening the door would make it difficult to generate an accurate demonstration dataset because we would need an accurate EEF controller to relate human to robot movements. In addition, we would not have any information on the amount of force applied during the demonstration. Guiding the robot using kinesthetic teaching would also rely on a good trajectory tracking controller. Because the tendons would not be loaded, any motor information would be useless, and we would still be missing force application data.

Using the SpaceMouse for teleoperation provides perfect data, but there is a learning curve involved. First, the operator must learn how to accurately control the SpaceMouse itself, and second, the operator must compensate for the errors from the open-loop EEF velocity controller. The solution to this could be to use a more intuitive demonstration device, as performed by Zhao et al. (2023) and Wu et al. (2024). However, once the user becomes familiar with the teleoperation system, the open-loop controller on the real system does not make the demonstration much more difficult. This is evident when comparing the average reward achieved when giving the expert demonstration, as shown in Figures 9, 10. In the simulation, the mean reward was 0.68, and on the real system, it was 0.66, even though the simulation environment used a directly driven robot without tendons and a closed-loop EEF controller. However, it should be noted that the user has better visual feedback when demonstrating on the real system because only a single camera view is provided in the simulation, which makes the depth vision less accurate. Although there is a difference in the number of time steps per episode recorded between the simulation and the real system, there is not a significant difference in wall clock time. In the simulation, each episode was 500 timesteps long at a step frequency of 20 Hz, which resulted in a recording time of 25 s. The real system has a step frequency of 5 Hz, and each episode is 130 timesteps long, resulting in a wall clock time of 26 s.

In both the simulation and real-world experiments, when providing the demonstration, no particular minimum quality level was enforced, except that each demonstration needed to reach the success state before the end of the episode. The results in Figures 9, 10 indicate that the TD7+Demo algorithm improves above the performance level of the demonstration, which makes the main purpose of the demonstrations to expose the policy to behaviors that reach the high-reward regions of the reward function. Therefore, the policy will likely reach the same performance level independent of the quality of the demonstrations, but the quality of the demonstrations will affect how fast the performance level is reached. It is important to note that the algorithm likely still works with some failed demonstrations in the dataset. However, if none of the demonstrations reach the success state, that is, there is no example data on how to get to the high-reward regions, the algorithm will not provide any real benefits over the default TD7 algorithm.

### 5.2 Learning in simulation

#### 5.2.1 Ablation study

The ablation study highlights the robustness of the proposed approach to the two newly introduced hyperparameters. For both Number of Demonstration Episodes in the Buffer and Percentage of Demonstration data in Learning Batch, all tested variations achieve a high reward at the end of the learning. This leads us to believe that the main benefit of using demonstration data is exposing the policy to the high-reward regions of the task. Therefore, even a few demonstrations drastically increase the performance, even though they do not cover the full range of variations. The results in Figure 7A are as expected: the more demonstrations are given, the more likely it is to have expert data on doing the task in all its variations. However, it is surprising that 50 demonstrations appear to perform worse overall than 10 and 20 demonstrations. However, the final scores for both 20 and 50 demonstrations are almost equal at 0.94 and 0.93, respectively. It is possible that the larger dataset does not add more valuable information and instead includes additional lower-performing demonstrations or greater variance in how the task is solved, guiding the policy in different directions and resulting in more unstable behavior in the early stages of learning. In general, we believe that the ideal number of demonstrations will depend on the amount of variance in the task. However, given that our approach combines imitation with exploration, it can achieve a high reward even if not all variations are represented in the dataset.

The results in Figure 7B show that all tested variations of the percentage of demonstration data per learning batch achieved a high reward at the end of learning. However, when considering the mean reward and variance, 50% clearly outperformed all other options, although this is likely to be dependent on the quality of the demonstration dataset. Figure 9B shows that the expert demonstrations given only achieved a mean reward of 0.67, which is substantially lower than what is achieved by the TD7+Demo algorithm of approximately 0.94. If the demonstration were closer to the optimum, using 75% of the demonstration data in each learning batch would likely perform much better.

We believe that the ideal percentage of demonstration data in each learning batch depends on the quality of the demonstrations. That is, if the demonstrations are mediocre in terms of reward, then having a high demonstration percentage in each learning batch could lead to the policy either taking a long time to reach a higher reward and/or landing in a local minimum. It should be noted that the ablation study does not evaluate all possible variations. For example, when we vary the number of demonstration episodes, the percentage of demonstration data in each learning batch is fixed to 50%. It is conceivable that this affects the results. That is, the higher the percentage of demonstration data in each learning batch, the more important the quality of the expert dataset in terms of both having enough demonstrations that cover most of the variance and the reward achieved.

#### 5.2.2 Offline learning

The TD7 Offline variant achieved a low reward on the door-opening task with the given demonstration datasets. In the original evaluation (Fujimoto et al., 2023), the D4RL dataset was used for the offline variant evaluation. Specifically, the “medium,” “medium-replay,” and “medium-expert” datasets were used for the HalfCheetah, Hopper, and Walker2d tasks. These datasets are crafted in a very specific way: online RL policies are trained to a certain point and then used to gather demonstration data. The “medium” dataset is generated by training an SAC policy to a medium performance level and then executing this partially trained policy to gather $10^{6}$ samples. The “medium-replay” dataset consists of recording all data observed while training to a medium performance level. The size of the dataset varies from task to task: in the Hopper environment, it has 200,920 steps; in the HalfCheetah environment, it has 101,000 steps; and in the Walker2d environment, it has 100,930 steps. The “medium-expert” combines data gathered from the medium dataset with data gathered by a SAC policy trained to expert performance and a randomly initialized policy. The dataset features a total of $2*10^{6}$ steps.

As previously described, RL algorithms tend to overestimate unseen state–action pairs, which is the reason why simply putting some expert demonstrations in the learning buffer will not result in a well-performing policy. The offline variant of the TD7 adds a behavioral cloning term to the policy training. However, the critic, which is also used to train the policy, still has an overestimation problem. Therefore, it makes sense that when we test TD7 Offline with only expert demonstrations, it performs poorly. The dataset used in the original evaluation was gathered by another RL policy at different learning states, resulting in a well-approximated distribution of the function space. However, this is not a realistic scenario, because if a policy must be trained online from scratch up to an expert state to gather data, then why not simply use that trained policy in the first place? It could be used for transfer learning, but we argue that the already trained policy would likely perform better.

With this in mind, the results we achieved during the experiments on different datasets that use human expert demonstrations, shown in Figure 8, make sense. Using only expert demonstrations, the reward at the end was close to zero. When additional data from a randomly initialized policy in the Random Expert dataset were added, TD7 Offline achieved a much higher performance. Adding additional data by partially replaying expert demonstrations combined with random movements in the Gradual Random Expert dataset resulted in a slightly higher final reward than the Random Expert dataset.

The authors believe that this shows that the offline variant relies on a dataset that contains a good approximation of the overall function distribution because, in our test, the dataset that explores the environment the most performs the best. When running the offline variant with the Gradual Random Expert dataset for a longer horizon, as shown in Figure 9A, we observe two effects: initially, the policy performance approaches the average expert demonstration score, after which the performance starts to degrade to almost zero. This is likely because the overestimation of the unseen state–action pairs increases over time, which at some point makes the critic term in the policy training overshadow the behavioral cloning term.

#### 5.2.3 Final evaluation

While the TD7+Demo algorithm achieved a high average reward of 0.94 with a low standard deviation within the first 200 exploration episodes, as shown in Figure 9B, it is slightly lower than the average reward of 0.98 after 10,000 episodes. Because the policy reliably reaches a value close to that after 400 episodes, one can consider whether it is worth the tradeoff of obtaining a slightly better performance level but spending double the time learning. For the experiments on the real hardware, we decided that a slight performance increase was not worth the extra time and therefore used 200 episodes instead. TD7 could achieve a similar performance level as TD7+Demo given enough time, but that outcome is not guaranteed, and the performance appears to be unstable, as demonstrated by the much lower average reward and large standard deviation. This is likely because the algorithm sometimes does not discover high-reward regions through random movements. It also remains close to zero in the beginning because the authors’ implementation does not start learning until a certain amount of data is gathered. Because TD7+Demo already starts with data in the demonstration buffer, we can start learning almost immediately.

### 5.3 Learning in the real world

The robot and door were in the same position throughout all experiments because manually moving the door and/or robot between episodes would be cumbersome. This can be solved by placing the GH360 on a mobile base. However, there is still variability between the different episodes resulting from the soft tendons between the motor and joint pulleys. The robot is controlled using an open-loop EEF velocity controller. Because the tendons make it so that the joint and motor positions are not correlated, the resulting movement, given the same goal velocity from the same joint angles, will result in different movements.

**FIGURE 11:**
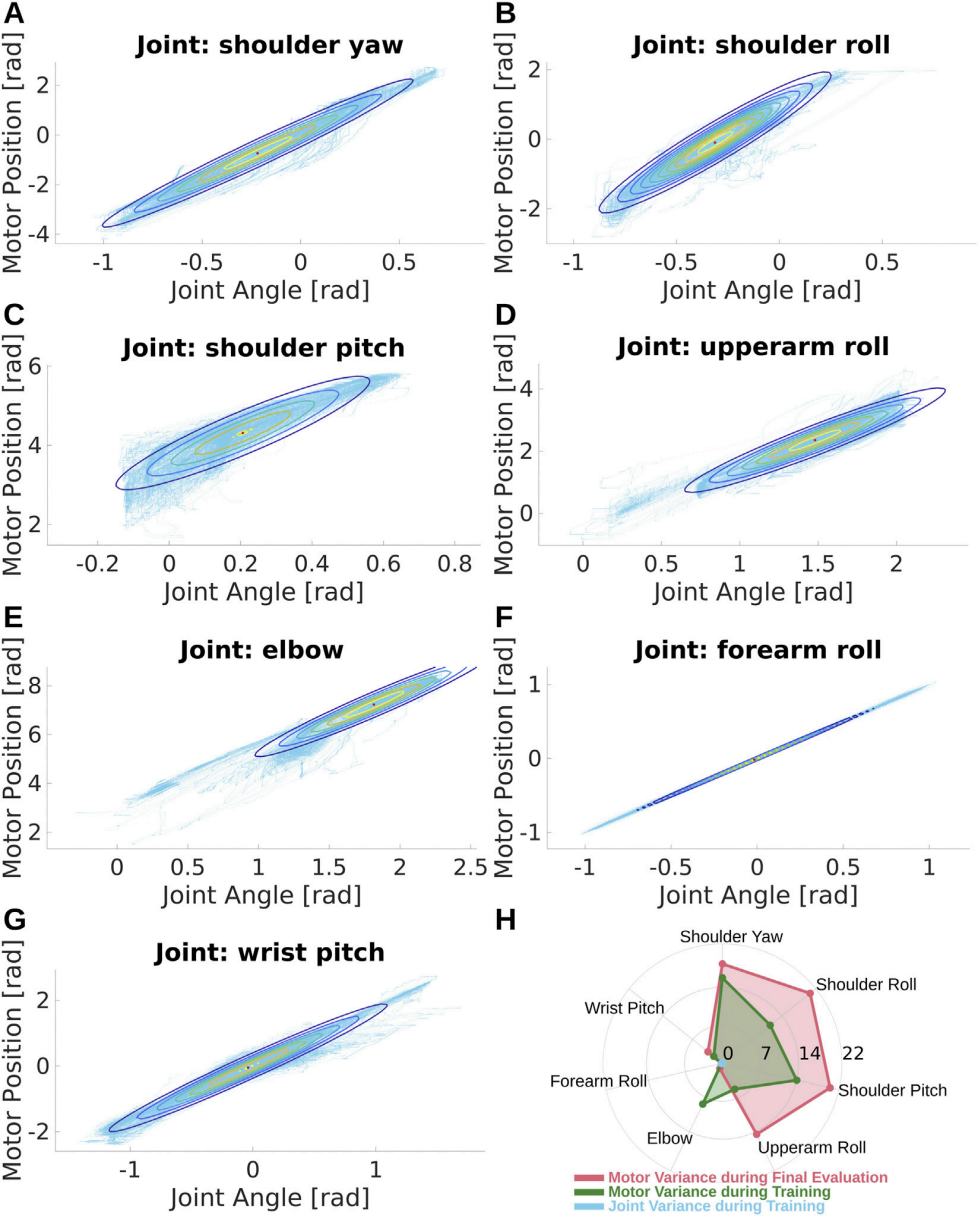
**(A–G)** Joint angle to motor position variance during one full learning run. **(H)** Motor angle $\left[rad*10^{3}\right]$ variance in reset position at episode start. The variance during the final evaluation is in relation to the mean motor positions during training, as this is what the policy has been trained on as well.

Figures 11A–G show the amount of variance between the motor and joint positions that occurred during a full learning run. Because the GH360 features one directly driven joint, namely, the forearm roll, we can see that a normal joint would show a clear line because the joint and motor position are the same, whereas in all the VSA joints, this is not the case. This is the reason why, during the final evaluation after learning, we perform special intermediate reset movements to expose the policy to as much variation as possible. Throughout the learning process, we used a closed-loop joint position controller to ensure that the robot started in the same kinematic configuration in every episode. However, the motor position still varies between different resets. Figure 11H shows the variance of the joint and motor angles during training and the motor angle variance during the final evaluation compared to the mean during training. No variance is observed in the joint angle because of the closed-loop position controller. No variance is observed in the forearm roll joint because it is directly driven by a motor.

The radar chart also shows that during the final evaluation, the trained policies are exposed to a larger variance through the intermediate reset position than during normal training, except for the elbow joint. The authors believe that there are two main reasons for this. First, the normal variance in that joint would be relatively low because the reset position of the joint (1.87 rad) is a position where the tendons are already stretched to a point where the rigid sleeve bears the majority of the load, which reduces the variance that comes from the soft inner filament of the tendon. Second, when the tendon is rolled up on the motor pulley for multiple rotations, the friction of the outer sleeve surfaces keeps the tendon in a static position on the pulley unless sufficient force is applied to overcome that friction. This occurs when the arm pushes or pulls something using the elbow joint. Because the intermediate reset positions for the final evaluation were free-space movements, the variance achieved specifically on the elbow joint was quite low.

One factor that has not been controlled for during real-world experiments is the friction between the hook and the door handle, as well as in the door latch mechanism itself. The hook is 3D printed using polylactic acid (PLA) and therefore has a smooth and slippery surface. The door handle also has a low-friction metal surface. We wrapped tape around the door handle, as shown in Figure 5A, making it more difficult to slip off. During learning, the tape degraded and therefore had to be replaced once. Similarly, because of the constant interaction with the door handle, friction inside the door latch mechanism increased over time, thereby increasing the force required to open the door. We lubricated the door twice throughout the experiments. In hindsight, the best practice would have been to replace the tape and lubricate the door before every learning run.

**FIGURE 12:**
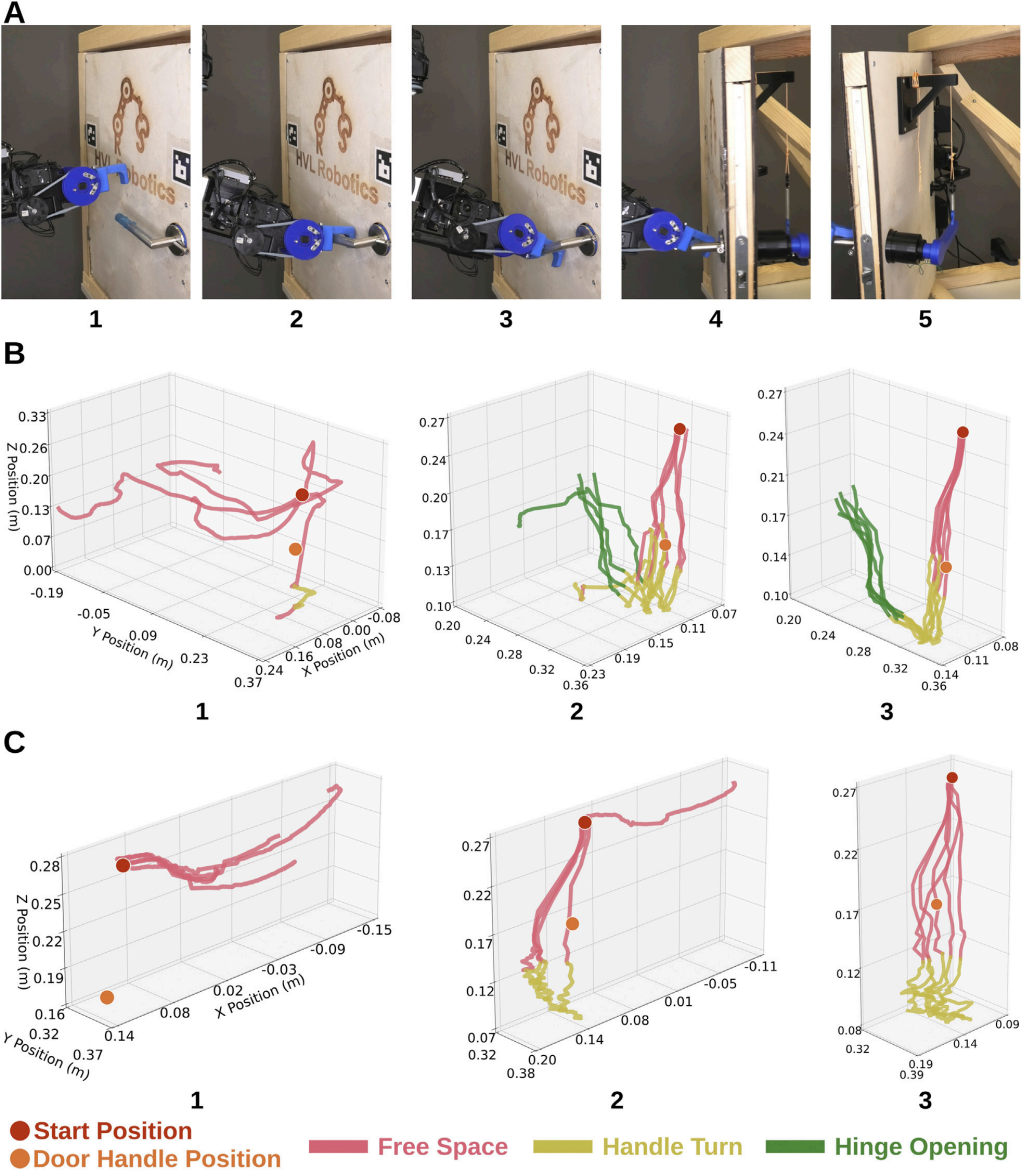
**(A)** Visualization of the phases of a successful door opening. (A.1) Reset/starting position. (A.2) The EEF of the robot is placed on the door handle. (A.3) The door handle is pushed down. (A.4) The door is pulled open. (A.5) The door has been successfully opened. **(B,C)** Evaluation trajectories from different evaluation periods. **(B)** TD7+Demo algorithm. **(C)** TD7 algorithm. Every ten exploration episodes, we perform an evaluation period consisting of five evaluation episodes. The trajectories shown are the first episode of the specified evaluation period. (1)Trajectories from evaluation periods 0–4. (2) Trajectories from evaluation periods 5–9. (3) Trajectories from evaluation periods 15–20.

During learning, we run evaluation periods to gather data on the performance of the current policy iteration without any randomness, which is normally added to encourage exploration. When learning on the real robot arm, each evaluation period consisted of five episodes. Evaluation period 0 is performed at the very beginning, that is, at timestep 0, after which we run an evaluation period after every 10 exploration episodes. Figure 12B,C show one of the five evaluation episode trajectories for the selected evaluation periods. The trajectories are colored to represent the different phases of the door-opening task, as shown in the picture sequence in Figure 12A. The Free Space phase represents the movement between the reset position and the hook being placed on the door handle (Figure 12 (A.1)–(A.2)). In the Handle Turn phase, the robot pushes down the door handle (Figure 12 (A.2)–(A.3)). Finally, the Hinge Opening phase represents the robot pulling the door open (Figure 12 (A.3)–(A.5)).

The results in Figure 10A show that the TD7 algorithm on the real system appears to get stuck in the middle reward region, never reaching a high reward in the given amount of time. This is similar to the results obtained during simulations, shown in Figure 9. When inspecting the evaluation trajectories in Figure 12 (C.3), we can see that it never discovers the rewards hidden behind opening the hinge and reaching the success state. The TD7+Demo, on the other hand, being guided by the expert demonstrations, started opening the hinge and reaching the success state as early as evaluation period 6 (7800 time steps), as shown in Figure 12 (B.2). The results in Figure 10A show that it begins to surpass the average reward achieved while giving the expert demonstration in evaluation period 7 (9100 time steps). The dip at evaluation period 13 (16,900 time steps) appears to be because the robot is trying to optimize the movement, leading to it slipping off the door handle, as can be seen in the videos provided as Supplementary Material. In general, the TD7+Demo algorithm opened the door from evaluation period 6, after which it improved both performance and robustness. This can be seen when comparing Figure 12 (B.2) and (B.3). Whereas (B.2) still has a relatively wide range of trajectories, in (B.3), the policy has found a reliable, high-performing trajectory.

While the door and robot are static, the results in Figure 10B show that when replaying a randomly chosen demonstration, the average success rate of opening the door is only 28% with a mean reward of 0.37. This shows that there is sufficient variance in the system such that simple replay does not work. That it is not simply that the demonstrations are not good can be seen when looking at the BC policy that has been trained on the same demonstrations and achieves a success rate of 74% with a mean reward of 0.62. This is not far from the 0.66, which is the mean reward achieved while giving the demonstrations. Part of the reason why the BC policy is not performing on par with the demonstrations could be that during evaluation, the policy is exposed to more variance, as shown in Figure 11, through the special resets. Another reason could be that the hyperparameters were not optimized but rather tuned empirically.

## 6 Conclusion and future work

In this study, we investigated the combination of exploration and imitation learning on an articulated soft robot arm with minimal task-specific engineering. We show that having soft tendons between the motors and the joint allows us to explore safely without pretraining the policy or requiring additional or external sensors but rather use demonstrations and onboard sensors to define the safety limits during learning, namely, joint angle and motor current limits. By combining state-of-the-art off-policy DRL learning with human demonstration, we can learn to open a door more efficiently than learning only from exploration and achieve a higher performance and robustness level compared to BC learning alone. We presented a thorough review of the problems that arise when learning contact-rich tasks on a real system and how the GH360 combined with our proposed approach handles them.

There are multiple directions for future improvements. The most obvious is to test our approach on other contact-rich tasks, such as wiping a surface, to fully evaluate the robustness of our proposed methodology, as well as contact-free tasks, such as pick and place, to evaluate the increase in complexity when learning tasks in which the passive compliance of the arm provides no advantage. Although the joints on the GH360 can change stiffness via co-contraction, we did not exploit that ability in this study but rather operated at a fixed stiffness level. Therefore, it would be of interest to investigate the impact of varying stiffness compared to the increase in learning complexity when increasing the action space. Adding a mobile platform to the GH360 robot would allow for a more realistic evaluation of the TD7+Demo algorithm in dynamic contact-rich environments. Another interesting future direction is to investigate how transferable our proposed algorithm is to other articulated soft robotic platforms, both in terms of learning from scratch and transferring trained policies.

Although we have limited the amount of task-specific engineering in our learning approach, some components are still not automated, namely, the reward function, automatic resetting of the environment, and using additional sensors for environmental observations. One approach to simplify this problem is to use a sparse reward function, as we then only need to infer from the demonstrations what is considered a success state and what is not, instead of learning a continuous function. The demonstrations could guide the RL policy to learn the task successfully using a technique called reward relabeling. In reward relabeling, if a success state is reached in an episode, all previous step rewards are changed from 0 to, for example, 0.5. As a result, the demonstrations can guide the RL policy to learn the task successfully.

Automating the resetting of the environment is a more complex problem. One approach could be to train a second policy to perform the opposite task. For example, while one policy learns to open a door, the other policy learns to close it. Finally, we equipped the door with additional sensors for environmental observation. However, the same information could be inferred using only the onboard camera of the robot. Other studies, such as Zhao et al. (2025), use ResNet50 to obtain a lower-dimensional feature map from the raw camera view.

## Data Availability

The datasets presented in this study can be found in online repositories. The names of the repository/repositories and accession number(s) can be found at https://github.com/LauEls/TD7/tree/frontiers_paper_2026.
