# Supplementary material for: Combining exploration and imitation in contact-rich task learning on an articulated soft robot arm
Source: Front Robot AI. 2026 Aug 5;13:1885625. doi: 10.3389/frobt.2026.1885625 (PMC13485553; doi:10.3389/frobt.2026.1885625)
Supplement: Supplementary file 1 [file Table1.pdf]

Joint Name	Special Reset									
	**1**	**2**	**3**	**4**	**5**	**6**	**7**	**8**	**9**	**10**
Shoulder Yaw	min	max	min	max	min	max	max	max	max	max
Shoulder Roll	min	max	max	min	min	max	min	max	max	max
Shoulder Pitch	max	min	max	min	max	max	max	max	max	max
Upperarm Roll	max	min	min	max	max	max	min	max	max	max
Elbow	min	min	max	max	min	min	min	max	max	max
Forearm Roll	min	max	min	max	min	min	min	max	max	max
Wrist Pitch	min	max	max	max	min	max	max	max	max	max

Table 1: Intermediate reset joint configurations for final evaluation.
