# Supplementary material for: Combining exploration and imitation in contact-rich task learning on an articulated soft robot arm
Source: Front Robot AI. 2026 Aug 5;13:1885625. doi: 10.3389/frobt.2026.1885625 (PMC13485553; doi:10.3389/frobt.2026.1885625)
Supplement: Supplementary file 2 [file Table2.pdf]

Joint Name	Joint Angle $[rad]$		Motor ID	Motor Current $[mA]$	
	**min**	**max**		**min**	**max**
Shoulder Yaw	-0.8218	0.6121	1	-588.0	245.28
			2	-1196.16	248.64
Shoulder Roll	-0.7622	0.2599	3	-631.68	1004.64
			4	-661.92	682.08
Shoulder Pitch	-0.1011	0.5305	5	-685.44	2388.96
			6	-178.08	836.64
Upperarm Roll	0.2902	1.9845	7	-1727.04	742.56
			8	-601.44	692.16
Elbow	0.1767	2.1778	9	-420.00	1992.48
			10	-624.96	1582.56
Forearm Roll	-0.9188	0.8759	11	-924.0	564.48
Wrist Pitch	-1.1277	1.3470	12	-682.08	534.24
			13	-487.2	678.72

Table 2: Robot limitations generated from demonstrations.
